# Targeting ATR offers multifaceted treatment strategies involving RAD51-mediated compensatory DNA repair in bladder cancer

**DOI:** 10.1186/s13046-025-03603-4

**Published:** 2025-12-12

**Authors:** Julia Pannhausen, Ahmed A. Chughtai, Cem-Louis Yüce, Michael K. Melzer, Yanchun Ma, Lancelot Seillier, Emiel P. C. van der Vorst, Geoffroy Andrieux, Julia Wirtz, Sophie Leypold, Mark P. Kühnel, Per Hoffmann, Stefanie Heilmann-Heimbach, Melanie Boerries, Alexander Kleger, Matthias Saar, Michael J. Eble, Danny D. Jonigk, Nadine T. Gaisa, Michael Rose

**Affiliations:** 1 Institute of Pathology, Uniklinik RWTH Aachen, Pauwelsstraße 30, Aachen, 52074 Germany; 2 Center for Integrated Oncology Aachen Bonn Cologne Duesseldorf (CIO ABCD), Aachen, 52074 Germany; 3 Department of Radiation Oncology, Uniklinik RWTH Aachen, Aachen, 52074 Germany; 4 Department of Urology, Ulm University Hospital, Ulm, 89081 Germany; 5 Institute for Molecular Oncology and Stem Cell Biology, Ulm University Hospital, Ulm, 89081 Germany; 6 Department of Internal Medicine I, Aachen-Maastricht Institute for Cardio-Renal Disease (AMICARE), Institute for Molecular Cardiovascular Research (IMCAR), University Hospital Aachen, Aachen, 52074 Germany; 7 Institute for Cardiovascular Prevention (IPEK), Ludwig-Maximilians-Universität Munich, Munich, 80336 Germany; 8 Institute of Medical Bioinformatics and Systems Medicine, Faculty of Medicine, Medical Center, University of Freiburg, University of Freiburg, Freiburg, 79110 Germany; 9 Institute of Human Genetics, School of Medicine & University Hospital Bonn, University of Bonn, Bonn, 53127 Germany; 10 German Cancer Consortium (DKTK), partner site Freiburg, a partnership between DKFZ, Medical Center – University of Freiburg, Freiburg, 79110 Germany; 11 Core Facility Organoids, Ulm University Hospital, Ulm, 89081 Germany; 12 Section for Interdisciplinary Pancreatology, Clinic for Internal Medicine I, Ulm University Hospital, Ulm, 89081 Germany; 13 Department of Urology and Pediatric Urology, Uniklinik RWTH Aachen, 52074 Aachen, Germany; 14 German Center for Lung Research, DZL, BREATH, Hanover, 30625 Germany; 15 Institute of Pathology, Ulm University Hospital, Albert-Einstein-Allee 23, Ulm, 89081 Germany

**Keywords:** Bladder cancer, Squamous cell carcinoma, DNA damage response, ATR, Ceralasertib, Homologous recombination repair, B02, RAD51, Cancer drug resistance

## Abstract

**Background:**

Muscle-invasive bladder cancer (MIBC) treatment depends on histological subtypes. While urothelial carcinoma (UC) benefits from novel therapies, options beyond radical cystectomy for rare subtypes such as squamous cell carcinoma (SCC) remain limited. Since we previously demonstrated ATR inhibitor (ATRi) enhanced radiation sensitivity in vitro*,* we aimed to further decipher the therapeutic impact of ATRi and compensatory pathways bypassing ATRi-resistance in patient-derived ex vivo cultures (PDCs).

**Methods:**

PDCs (p-SCC, p-UC, *n* = 6) were established and characterized by immunohistochemistry, qPCR, and whole-exome sequencing. Independent ATRi-resistant cell models (p-SCC^ATRi^) were generated through long-term ATRi treatment (Ceralasertib) and characterized by multi-dimensional profiling. Drug responses were analyzed via cell viability (IC50) and clonogenic survival assays ± ionizing radiation (IR). DNA repair capacity was measured via γH2AX immunofluorescence, comet assays, qPCR, and immunoblotting. ATR siRNA knockdown and ATRi short-term studies validated the ATR-RAD51 axis. RAD51 inhibitor (RAD51i) B02 was tested in p-SCC^ATRi^ by cell cycle analysis and *in ovo* tumor growth of chorioallantoic membrane (CAM) xenografts, complemented by apoptosis staining.

**Results:**

ATRi treatment sensitized cells to IR, reducing IC50 values up to 2.5-fold (at 8 Gy: 0.52 µM in SCC, 0.82 µM in UC). Clonogenic assays and γH2AX staining confirmed impaired DNA repair (γH2AX foci at 8 Gy: 11-fold in SCC, 15-fold in UC). In resistant p-SCC^ATRi^ models, ATRi-adaptation triggered various compensatory and potentially epigenetic regulated DNA repair pathways, particularly homologous recombination (HR) repair involving genes like *BRCA1* and *RAD51*. Downstream consequences of functional ATR loss also affected non-DNA repair processes such as cell cycle, chromatin reorganization, and immunomodulation. As a therapeutic strategy, RAD51i overcame resistance by lowering IC50 by 40–80%, increasing DNA damage (2.2-fold γH2AX foci), and inducing G2/M arrest (2.4-fold). Finally, *in ovo*, RAD51i significantly induced apoptosis impairing tumor growth in p-SCC^ATRi^ xenografts by up to 37%.

**Conclusion:**

Our results propose ATR as a promising target in bladder cancer by (1) enhancing radio-sensitivity through classical ATR inhibition and (2) exploiting resistant ATRi-adaptation as a vulnerability by targeting compensatory HR repair reliance through RAD51 inhibition. These findings on the ATR–HR axis suggest novel strategies to improve bladder cancer treatment and addressing therapy resistance.

**Supplementary Information:**

The online version contains supplementary material available at 10.1186/s13046-025-03603-4.

## Introduction

Bladder cancer (BLCA) is a histologically heterogeneous disease with distinct subtypes [1–3]. Urothelial carcinoma (UC) is the most common subtype, whereas squamous cell carcinoma (SCC) represents a rarer variant with distinct biology, prognosis, and worse treatment response [3]. Standard therapy for muscle-invasive bladder cancer (MIBC) is radical cystectomy (RC), while trimodality therapy (TMT) with maximal TURBT plus chemoradiation has emerged as an evidence-based bladder-preserving alternative for selected MIBC patients [4]. Studies show TMT achieves outcomes comparable to RC in node-negative disease [5–9], and UK data suggest similar survival in clinically node-positive cases [10]. EAU and NCCN guidelines [11, 12] now recommend TMT as an evidence-based option for eligible patients and not only for those unfit for surgery. Beyond bladder preservation, radiotherapy is gaining interest in broader treatment settings. A recent randomized trial demonstrated benefits of adjuvant radiotherapy after cystectomy and systemic therapy in high-risk patients [13], underscoring its evolving role in BLCA management. This is particularly relevant for SCC, which shows limited response to chemotherapy and cisplatin resistance [14, 15], lacks effective targeted therapies and robust clinical trial data, and relies on RC as the primary treatment [16].

These contribute to suboptimal clinical outcomes and poor prognosis in SCC [17], highlighting the need for improved therapies. Therapeutic development for SCC is further hindered by the limited availability of representative models. Although several BLCA cell cultures exhibit basal/squamous-like characteristics [18], SCaBER remains the only widely accepted in vitro model closely reflecting SCC biology [19].

Previously, we investigated radio-sensitization strategies in UC (VMCUB1, J82) and SCC (SCaBER) cells by pharmacologically targeting the DNA damage response (DDR) pathway [20]. DDR mechanisms are essential for genomic stability and repair of genotoxic stress [21] and their therapeutic exploitation has already been successfully translated into clinical application [21, 22]. One prominent example is the use of poly(ADP-ribose) polymerase (PARP) inhibitors, such as Olaparib, which induce synthetic lethality in tumors with homologous recombination (HR) repair defects [23, 24]. Building on this concept, a new generation of DDR inhibitors has emerged [21, 25], including ataxia telangiectasia and Rad3-related kinase (ATR) inhibitors that target a key regulator of replication stress response [22, 26].

ATR facilitates DNA repair via the BRCA1–RAD51 axis and regulates cell cycle checkpoints through CHK1 signaling [27]. Among ATR inhibitors (ATRi), Ceralasertib (AZD6738) [28] has shown efficacy in preclinical models and clinical trials across various tumor types. Notably, Phase II studies have shown benefit when combined with the PARP inhibitor Olaparib, particularly in HR-deficient cancers such as ovarian cancer (NCT03462342) [29], breast cancer (CRUK/15/010) [30], and osteosarcoma (NCT04417062) [31]. In addition, a Phase III trial is currently evaluating Ceralasertib in combination with the immune checkpoint inhibitor durvalumab in non-small cell lung cancer (NCT05450692) [32]. Ceralasertib has also been investigated as a radio-sensitizer in preclinical models of melanoma [33], colorectal [33, 34], hepatocellular [35], and lung carcinoma [34, 36], as well as in SCC of head and neck, ovary, and oral cavity [34]. Sensitivity to ATR inhibition has been linked to expression of *CCNE1* [37, 38], *RAS* [39], *MYC* [40], or *CDC25A* [41], with synthetic-lethal interactions proposed for *ARID1A* [21], *APOBEC* [42], *ATM* [43], and *DNA-PK* [22].

Despite the promising potential of DDR inhibitors, resistance driven by bypass pathways limits their long-term efficacy [44]. While PARP inhibitor resistance is well studied, mechanisms of ATRi-resistance remain less defined. Emerging evidence highlights resistance-associated alterations in *CCNE1*,* CDK2*,* MYC* [45, 46], *FOXM1*,* ECT2* [47], *CDC25A/B* [48], and *CDK8/CCNC* [49], all of which may reduce tumor dependency on ATR signaling [50, 51]. These adaptations activate compensatory pathways to counterbalance independence from ATR-mediated DNA repair and still maintain genomic stability [50], creating potential vulnerabilities exploitable by targeting those compensations [46, 50].

For genomic stability and cell survival, ATR and homologous recombination (HR) repair are essential [52]. HR ensures high-fidelity repair of DNA double-strand breaks, particularly during replication or after fork collapse through coordinated action of BRCA1 and BRCA2, which mediate end resection, RAD51 filament formation, and strand invasion [53]. These factors work in close coordination with ATR signaling, which detects replication-associated damage and stabilizes stalled replication forks to create a permissive environment for HR repair [22]. Notably, ATRi show enhanced efficacy in HR-deficient tumors [54], highlighting the functional and therapeutic interplay between ATR and HR repair.

In this study, we aimed to (1) validate the radio-sensitizing effects of the ATRi Ceralasertib from our previous study by using patient-derived SCC (p-SCC) and UC (p-UC) ex vivo cultures, (2) characterize compensatory DNA repair mechanisms arising from resistant ATRi-adaptation, and (3) evaluate the efficacy of targeted compensatory pathways by inhibition of HR repair pathway to overcome ATRi induced resistance and to bypass mechanisms of *ATR* mutated BLCA.

## Methods

### Small molecule inhibitors

The ATR inhibitor (ATRi) Ceralasertib (AZD6738) was purchased from Selleck Chemicals (product code: S7693). The RAD51 inhibitor (RAD51i) B02 was purchased from Sigma-Aldrich (product code: 553525). Unless otherwise specified, standard inhibitor concentrations were 1 µM for ATRi and 5 µM RAD51i.

### Patient-derived primary bladder cancer cultures

2D patient-derived ex vivo cell cultures were isolated and cultured as described previously [55] using fresh cystectomy material from BLCA patients directly after surgery at Uniklinik RWTH Aachen. Corresponding tissue samples were cryopreserved for mutational and transcriptomic analyses. In total, tumor tissue from 33 cystectomies was used to establish patient-derived ex vivo cell cultures. For reproducibility, only cultures with long-term growth (passages >10) and displaying original tumor characteristics were included in subsequent analyses, yielding a cohort of bladder squamous cell carcinomas (SCCs) (*n* = 3: p-SCC1, p-SCC2, p-SCC3) and urothelial carcinomas (UCs) (*n* = 3: p-UC1, p-UC2, p-UC3). Experiments were in accordance with the regulations of the Institutional Review Board (IRB)-approved protocols of the Medical Faculty of RWTH Aachen University (EK 268/21, EK 206/09 study number 199 and 311) and written patient consent was available through the RWTH centralized Biomaterial Bank (RWTH cBMB).

### ATRi-resistant models

Dysfunctional ATR models of p-SCCs (p-SCC1^ATRi^, p-SCC2^ATRi^, p-SCC3^ATRi^) were developed as described [56] through long-term treatment (>six months) with an ATR inhibitor (Ceralasertib, AZD6738). Ceralasertib concentration was gradually increased from 0.05 µM to a final concentration of 0.60 µM over the adaptation period. This final concentration (0.60 µM) was subsequently maintained in the culture medium during routine cell passaging to preserve the ATRi-adapted phenotype and used as the background condition for all “adapted” experiments. Unless otherwise stated, all cell culture experiments were performed independently at least three times.

For 3D culturing, 3.0 × 10^4^ cells (p-UCs, p-SCCs, p-SCCs^ATRi^) were seeded in 50 µL Ultimatrix (BME001-05, Cultrex) in a 24-well plate and propagated as described [57]. To maintain the adapted phenotype, p-SCCs^ATRi^ were continuously exposed to 0.60 µM ATR inhibitor. After passaging, 500 nM ROCK inhibitor (Y‑27632, Stemcell Technologies) was added to the culture medium.

### Transient siRNA *ATR* knockdown

p-SCC1 wild-type cells were seeded in 6-well plates at 2.0 × 10^5^ cells/cm^2^. After attachment, cells were transfected with 12 µL HiPerfect (301705, Qiagen) and 12 µL ATR-specific siRNA (SR319441, 5 µM, OriGene Technologies) or non-targeting control siRNA (SR30004, 5 µM, OriGene Technologies) resulting in a final siRNA concentration of 30 nM. Cells were harvested at 48 h, 96 h, and 144 h post-transfection for RNA and protein extraction.

### Short-term ATR inhibition

For pharmacologic ATR inhibition, p-SCC1 wild-type cells were seeded in 6-well plates at 2.5 × 10^4^ cells/cm^2^. After attachment, cells were treated with 1 µM Ceralasertib (AZD6738) and incubated for 24–96 h. Cells were subsequently harvested for RNA and protein extraction.

### TCGA BLCA dataset

Public BLCA datasets from The Cancer Genome Atlas (TCGA) Network [58] were used to validate *ATR*-, *CHEK1*, and *HR*-deficient specific downstream comprising mutation data of overall *n* = 1,527 samples from *n* = 1,244 patients [59]. Data were accessed and visualized by using the cBio Cancer Genomics Portal (https://cbioportal.org) [60].

### Immunohistochemistry (IHC)

For histology, formalin-fixed paraffin-embedded (FFPE) sections were stained with hematoxylin and eosin (HE). IHC was performed with anti-cleaved caspase-3 (1:400, #9661, Cell Signaling) using the Dako EnVision FLEX system (pH 9, K8000; Agilent), DAB + substrate (K3468, Agilent), and hematoxylin counterstain [61]. Staining for anti-CK5/6 (1:100, M7237, DAKO), anti-CK7 (1:100, M7018, DAKO), anti-CK20 (1:50, M7019, DAKO), anti-GATA3 (1:250, CM 405 A, BioCare Medical), anti-p63 (1:50, M7317, DAKO) and anti-UPK2 (1:100, ACI3051C, BioCare Medical) was conducted manually or on an Autostainer (Thermo Fisher Scientific, 360-2D) as described [62]. Protein expression was semi-quantitatively assessed using the immunoreactive score (IRS) by Remmele and Stegner [63].

### DNA extraction

For cell culture DNA isolation, cells were pelleted and processed with the QIAamp^®^ DNA Mini Kit (51304, Qiagen) following the manufacturer’s instructions. Cryopreserved bladder tissue (tumor and adjacent muscle) was manually microdissected and processed using the Maxwell^®^ 16 LEV Blood DNA Kit (AS1290, Promega). DNA quantity and purity were assessed on a NanoDrop^®^ ND-1000 spectrophotometer (Thermo Fisher Scientific).

### Whole exome sequencing (WES)

Whole-exome sequencing (WES) was performed at the Cologne Center for Genomics (CCG, Cologne, Germany) on 21 samples: patient-derived cell cultures (*n* = 3 p-SCCs, *n* = 3 p-UCs), ATRi-resistant cell lines (*n* = 3 p-SCC^ATRi^), corresponding adjacent tumor tissues (*n* = 3 SCCs, *n* = 3 UCs), and matched adjacent muscle tissues (*n* = 6) as normal controls to exclude germline variants in a blinded automated procedure. Libraries were prepared using the Agilent SureSelectXT HS Human All Exon V8 enrichment kit and XTHS2 protocol with Unique Molecular Identifiers (UMIs) for error correction and PCR duplicate removal. WES was performed on an Illumina NovaSeq 6000 (2 × 100 bp, paired-end). Delivered FASTQ data were processed using the *nf-core sarek* pipeline somatic workflow (v3.1) [64, 65] against the GRCh38 reference genome. SNV and InDel calling was performed using Mutect2 (v4.5.0.0), copy-number alterations were called using cnvkit (v0.9.11) and microsatellite status was assessed using msisensor-pro (v1.2.0). Mutation calls were annotated with the Ensembl Variant Effect Predictor (cache version release 112) [66] and post-processed using an in-house pipeline [67]: Annotated variant calls were filtered for ≥ 10× coverage, ≥ 5% allelic fraction and for less than 2% variant allele frequency in the gnomAD v4.1.1 global population. Tumor mutational burden was calculated using the filtered mutational calls using the in-house pipeline based on the Agilent SureSelectXT HS Human All Exon V8 target regions. Copy-number alterations were filtered for segments with a size ≥ 50 kbp. Reactome pathways, associated with DNA repair, were analyzed for their enrichment based on mutations referring to the reactome pathway browser (v.3.7) [68].

### Methylome analysis (EPIC-Array)

Genome-wide DNA methylation profiling was performed using the Infinium MethylationEPIC BeadChip v2.0 (Illumina) [69] in collaboration with Life&Brain GmbH. Isolated DNA was bisulfite-converted and processed. IDAT-files were imported into R and analyzed with the *minfi* package (v1.54.1) [70]. After quality control and SWAN normalization [71] non-informative CpG sites (e.g., sex-chromosomal or cross-reactive probes) were excluded. Differential methylation between control and resistant samples was assessed using the *limma* package (v3.65.4) [72], reporting log2 fold change (logFC), p-values, and Benjamini–Hochberg adjusted p-values. Functional annotation of CpGs was performed via *biomaRt* (v2.64.0) [73] using Ensembl (GRCh38) [74], including mapping to transcription start sites, transcript lengths, and UTRs. Gene set enrichment was performed with clusterProfiler (v4.16.0) [75] and MSigDB (v1.16.0) [76] as reference database. Gene Ontology (GO) enrichment analysis was performed using PANTHER Classification System [77].

### RNA extraction

For RNA isolation from cultured cells, lysis was performed with RNA lysis buffer (740906.125, Macherey‑Nagel) followed by extraction using the NucleoSpin^®^ RNA Plus Kit (740984.50, Macherey‑Nagel) according to the manufacturer’s instructions. For cryopreserved bladder tissue (SCCs *n* = 3, UCs *n* = 3), tissue was manually microdissected and RNA extracted with the Maxwell^®^ 16 LEV simplyRNA Tissue Kit (AS1280, Promega). RNA concentration was measured using a NanoDrop^®^ ND-1000 spectrophotometer (Thermo Fisher Scientific).

### cDNA synthesis and quantitative real-time reverse transcription PCR

cDNA synthesis was performed using the Reverse Transcription System Kit (A3500, Promega) with 1 µg total RNA on a Bio-Rad C1000 Touch Thermal Cycler. Mastermix components and cycling conditions are detailed in Supplementary Table S1. mRNA expression was analyzed by real-time qPCR (RT-qPCR) on a CFX96 Touch system (Bio-Rad) using iQ™ SYBR^®^ Green Supermix (1725125, Bio-Rad) and Power SYBR™ Green PCR Master Mix (4367659, Thermo Fisher Scientific). Samples were measured in triplicate. Relative expression levels were calculated via the 2^−∆∆Ct^ method using *GAPDH* as reference housekeeping gene. Primer sequences and PCR conditions are listed in Supplementary Table S1.

### Transcriptome sequencing (RNA-Seq)

RNA sequencing (RNA-Seq) was performed at the Cologne Center for Genomics (CCG, Cologne, Germany) on two dysfunctional ATR models (p-SCC1^ATRi^, p-SCC3^ATRi^). Libraries were prepared using the Illumina TruSeq Stranded mRNA with ERCC RNA Spike-In Mix 1 for quality control and normalization. Poly(A) + mRNA was purified, fragmented, and reverse-transcribed using random primers. After second-strand synthesis, end repair, A-tailing, and adapter ligation, libraries were PCR-amplified (15 cycles), quality-checked (Agilent TapeStation), quantified (KAPA Library Quantification Kit), and pooled equimolarly. Sequencing was performed on an Illumina NovaSeq 6000 (2 × 100 bp, paired-end), generating ~ 60 million clusters per sample. Paired-end reads were trimmed to remove adapter content and low quality bases using Trimmomatic (v0.39) [78]. Reads were aligned to the human genome hg38 and reads-per-gene were quantified using STAR (v2.7.11b) [79]. Downstream analysis was done in R (v4.4.1) [80]. Differential analysis was performed with edgeR (v4.2.2) [81] accounting for library size and TMM normalization. Gene-set enrichment analysis (GSEA) was performed with clusterProfiler (v4.16.0) [75] and MSigDB (v1.16.0) [76] as reference database. For gene-set variation analysis (GSVA), normalized gene expression data were used as input to GSVA R package (v2.2.0) [82] to obtain per-sample pathway activity scores. MSigDB (v1.16.0) [76] gene sets were used. GSVA was run with method = “gsva”, kcdf = “Gaussian”, and default settings otherwise. Differential pathway activity between groups was tested on the GSVA score matrix using limma. A p-value < 0.05 was considered statistically significant.

### Ionizing radiation (IR) source and setup

Irradiation was performed using a medical patient linear accelerator (LINAC). Cell culture plates with cells and media were irradiated using 6 MV X-rays, positioned at a maximum dose depth of ~ 1.5 cm. The table height was adjusted to ensure a 100 cm source-to-surface distance. Additionally, 3 cm of water-equivalent material was placed opposite the plates to allow backscatter.

### Kinase activity profiling

Cells were seeded and harvested 30 min after 2 Gy treatment by washing with ice-cold PBS and lysed on ice for 15 min using M-PER Mammalian Protein Extraction Reagent supplemented with Halt Phosphatase and EDTA-free Halt Protease Inhibitor Cocktails (1:100 each; Thermo Fisher Scientific). Lysates were centrifuged at 16,000 × g for 15 min at 4 °C, and protein concentrations were determined using the Pierce™ Bradford Plus Protein Assay (Thermo Fisher Scientific).

Kinase profiles were determined using PamChip^®^ peptide tyrosine kinase (PTK, 196 phospho-sites) and serine/threonine kinase (STK, 144 phospho-sites) microarrays on the PamStation^®^12 system (PamGene International) with phosphorylation detected by FITC-labeled anti-phosphotyrosine or anti-phospho-Ser/Thr antibodies. For PTK, 9 µg protein (*n* = 3 per condition) was processed with PamGene protocols and reagents. 40 µL PTK Basic Mix were loaded per array, and assays were run for 94 cycles. Images were captured via PamStation^®^12 CCD at kinetic cycles 32–93 (10, 50, 200 ms) and end-level cycles (10–200 ms). For STK assays, 1 µg protein and 400 µM ATP were applied (*n* = 3 per condition) with antibody mix, incubated 1 h at 30 °C under continuous flow, followed by FITC-conjugated secondary antibody. Imaging used an LED-based system. Spot intensities were background-corrected and quantified in BioNavigator v6.3 (PamGene). Upstream Kinase Analysis (UKA) [83] ranked kinases by combined specificity (peptide–kinase linkages) and sensitivity (treatment–control differences) scores. Pathway enrichment was performed on differentially expressed kinases (median final scores >1.2, adj. p-value < 0.05) using R packages clusterProfiler (v4.0) [84], DOSE [85] and ReactomePA (v1.44.0) [86].

### Immunoblotting

Protein isolation, SDS-PAGE, and immunoblotting were conducted as previously described [20]. Membranes were blocked in 5% BSA for 1 h and incubated overnight at 4 °C with primary antibodies: anti-GAPDH (1:1000, #2118, Cell Signaling Technology), anti-Vinculin (1:1000, #13901, Cell Signaling Technology), anti-Artemis (1:1000, PA5-102814, Thermo Fisher Scientific), anti-Artemis pSer516 (1:250, ab138411, Abcam), anti-DNA-PK (1:1000, #4062, Cell Signaling Technology), anti-DNA-PK pSer2056 (1:1000, P00645, BosterBio), anti-BRCA1 (1:500, OP92, Merck), anti-CHK1 (1:1000, #2360, Cell Signaling Technology), anti-CHK1 pSer317 (1:250, #2344, Cell Signaling Technology), or anti-RAD51 (1:500, sc-8349, Santa Cruz Biotechnology). Secondary antibodies HRP-linked goat anti-rabbit IgG (1:5000, #7074, Cell Signaling Technology) and HRP-linked horse anti-mouse IgG (1:5000, PI-2000, Vector Laboratories) were applied for 1 h at room temperature.

### Cell growth assay

Cell count assays were used to determine cell growth. A seeding density of 6.0 × 10^3^ cells/cm^2^ was used per well. Every 24 h over a total period of 96 h, cells were harvested and counted using the CASY Cell Counter (OLS OMNI Life Science). The number of viable cells was recorded. Doubling time was calculated by plotting the natural logarithm of mean cell counts over time and determining the growth rate constant *k* as the slope of the linear regression. Proliferation rates (% increase per day) were estimated by doubling times to quantitatively describe cell growth dynamics.

### Cell viability assays

For 2D cultures, cells were seeded in 96-well plates at 1.6 × 10^4^ cells/cm^2^. After attachment, cells were treated in triplicates with a range of concentrations of Ceralasertib (0.01–20 µM) or B02 (0.01–100 µM) 1 h prior to IR (0, 2, or 8 Gy). Controls included untreated/non-irradiated and untreated/irradiated cells. After 72 h, cell viability was assessed by XTT assay (Roche, 11465015001) according to manufacturer’s instructions. For 3D cultures, drug treatment was performed as previously described [57]. Briefly, 5.0 × 10^2^ cells/well were seeded in 1 µL Ultimatrix in a 384-well plate using a MANTIS pipetting robot (Formulatrix). After polymerization of Ultimatrix, culture media containing 500 nM Rho-associated kinase inhibitor (Y-27632, Miltenyi Biotec) was added. After 24 h, cells were treated in triplicate with a range of Ceralasertib (0.01–20 µM) or B02 (0.01–100 µM). Controls included untreated cells. After 96 h, cell viability was assessed using CellTiter-Glo^®^ 3D (Promega, G9682). Luminescence was measured using a Tecan Infinite M200 Pro (Tecan). Drug response curves and IC50 calculation were performed as described before [61].

### Clonogenic survival assay

Cells were cultured overnight at densities adjusted according to IR doses (0 Gy: 3.1 × 10^4^ cells/cm^2^, 2 Gy: 3.4 × 10^4^ cells/cm^2^, 8 Gy: 4.2 × 10^4^ cells/cm^2^) and treated with inhibitors 1 h prior to IR. Controls included untreated/non-irradiated and untreated/irradiated cells. After 14 days, cells were stained with 0.25% crystal violet (Merck, 42555), and clonogenic survival quantified densitometrically, as previously described [20].

### Neutral comet assay

Cells were seeded at 4.3 × 10^4^ cells/cm^2^, allowed to attach overnight and treated with inhibitors 1 h prior to IR. Controls included untreated/non-irradiated and untreated/irradiated cells. At 0, 1, 3, and 6 h post-IR, cells were processed using the CometAssay^®^ Kit (R&D Systems) according to manufacturer’s instructions and as described [20]. DNA damage was quantified by tail moment using the OpenComet plugin for ImageJ (NIH) for automated analysis.

### Immunofluorescence (IF) staining for double-strand break (DSB) detection

Cells were seeded at 0.3 × 10^4^ − 1.1 × 10^4^ cells/cm^2^, attached overnight and treated with inhibitors 1 h prior to IR. Controls were untreated/non-irradiated or untreated/irradiated. At defined post-IR time points, cells were fixed and stained for IF as described [87], using anti-γH2AX (1:500, #05–636, Merck Millipore) and goat anti-rabbit IgG Alexa Fluor™ 488 (1:1000, A-11008, Thermo Fisher Scientific). Nuclei were counterstained with DAPI and mounted with Epredia™ Immu-Mount™ (9990402, Epredia). Confocal images were acquired with a Zeiss LSM 710 (Oberkochen, Germany) using a Plan-Apochromat 20×/0.8 NA objective and 405/488 nm lasers. Image acquisition was performed using ZEN Black software, version 2.3 SP1 FP3 (64-bit). Images were quantified in ImageJ (NIH), analyzing ≥ 50 cells per cell culture and condition.

### Fluorescence-activated cell sorting (FACS)

For cell cycle analysis, cells were seeded at 1.0 × 10^4^ cells/cm^2^, attached overnight and treated with inhibitors 1 h prior to IR. Cells were collected at specific post-IR time points and fixed in 70% ethanol at 4°C. DNA was stained with propidium iodide solution (50 µg/mL PI (Thermo Fisher Scientific, P3566), 100 µg/mL RNase, 1% BSA) for 30 min in the dark. The BD FACSCanto™ II (BD Biosciences) system with 3 lasers (405 nm, 488 nm, 633 nm) and a configuration for 8 fluorescent parameters (2–4−2) was used for flow cytometric analysis and the BD FACSDIVA Software Version 9.0.1 was used for data acquisition. At least 1.0 × 10^5^ cells were counted and cell cycle phases were gated and quantified using FlowJo™ v10.10.0 (BD Life Sciences).

### Chorioallantoic membrane assay (CAM)

Chicken eggs were obtained from Campus Frankenforst, Faculty of Agriculture, University of Bonn (Königswinter, Germany). CAM assay followed established procedures [88]. Initially, 2 × 10^6^ cells were seeded on embryonic day (ED) 8. By ED11 and ED13, visible xenografts had formed and were treated with either control (DMSO), a single inhibitor, or a combination of both inhibitors. Tumors were harvested at ED15 to measure tumor weight. Xenografts were processed into FFPE blocks for IHC staining.

### Statistical data acquisition

Statistical analysis used IBM SPSS 29.0.0.0 (SPSS) and GraphPad Prism 10 (GraphPad). Non-parametric Mann-Whitney U tests compared two groups, while Kruskal–Wallis and Dunn’s multiple-comparison tests compared multiple groups. Fisher’s exact tests and two-sided log-rank tests assessed clinicopathological correlations. Kaplan-Meier curves and log-rank tests evaluated overall survival (OS) and relapse-free survival (RFS). The following symbols indicate two-sided p-values: *p < 0.05, **p < 0.01, ***p < 0.001.

## Results

### Comprehensive characterization of patient-derived ex vivo cultures for SCC and UC as models reflecting tumor biology

**Fig. 1:**
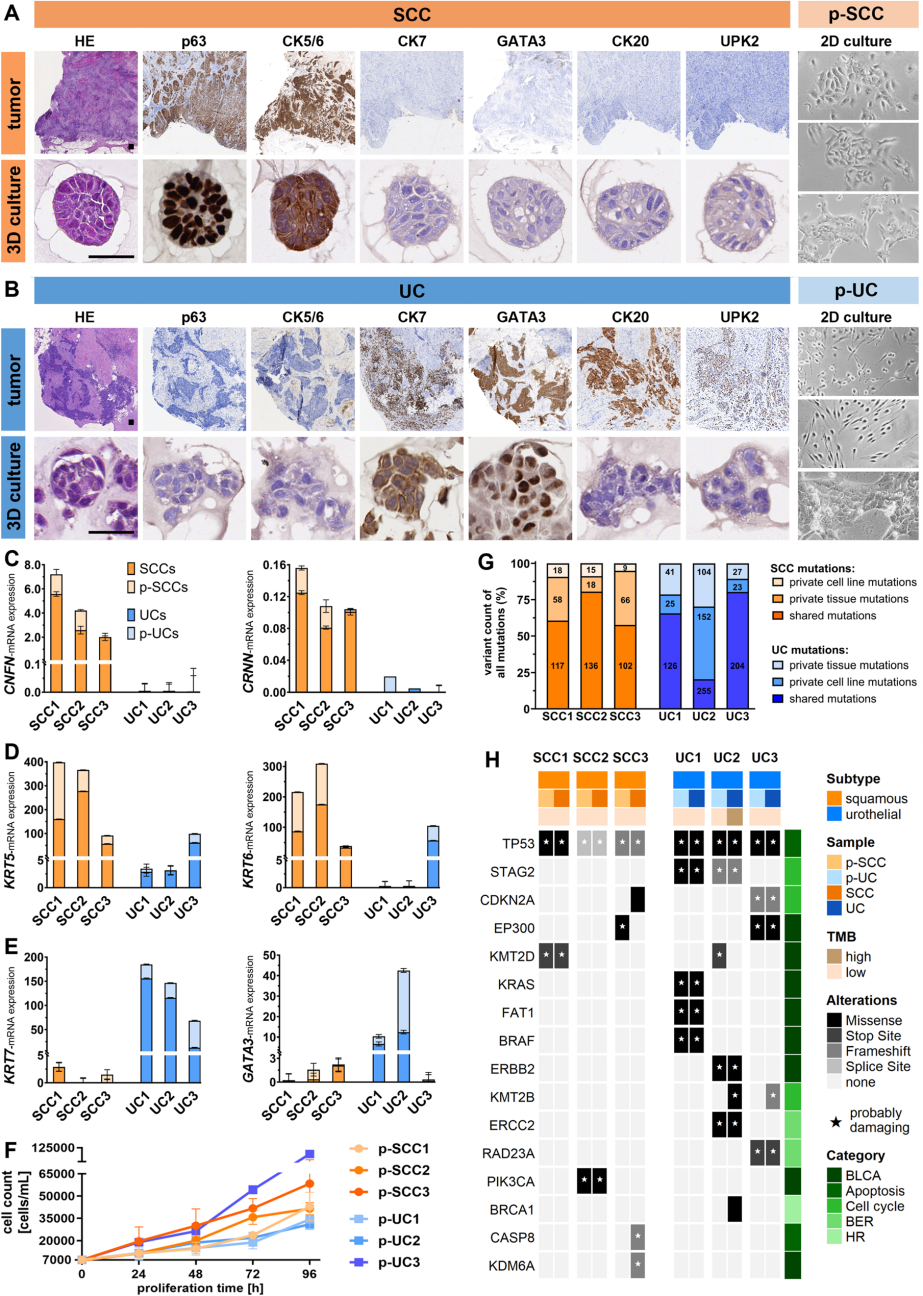
Comprehensive characterization of SCC and UC and adjacent patient-derived ex vivo cell cultures. **A-B** Histological and immunohistochemical characterization of tumor tissue, 3D patient-derived spheroid cultures, and light microscopy of 2D patient-derived cell cultures from squamous cell carcinoma (SCC) and urothelial carcinoma (UC). **A** SCC panels: representative sections of original SCC tissue and corresponding patient-derived 3D culture stained with HE, p63, cytokeratin 5/6 (CK5/6), cytokeratin 7 (CK7), GATA3, cytokeratin 20 (CK20) and Uroplakin 2 (UPK2) (scale bar: 100 μm for tumor; 25 μm for 3D culture). p-SCC panel: light microscopy of three 2D cultures (p-SCC1, p-SCC2, p-SCC3) (scale bar: 25 μm). **B **UC panels: representative sections of original UC tissue and corresponding patient-derived 3D cultures, as shown for SCC in (**A**). p-UC panel: light microscopy of three 2D cultures (p-UC1, p-UC2, p-UC3) (scale bar: 25 μm). **C-E** Confirmation of ex vivo cell culture character of p-SCCs and p-UCs based on qPCR marker expression of (**C**) *CNFN-* and *CRNN-,* (**D**) *KRT5-* and *KRT6-*, (**E**) *KRT7-* and *GATA3-*mRNA compared to their respective primary tumors. *GAPDH* served as a control. **F** Cell count assay of p-SCCs and p-UCs over 24–96 h. **G-H** Genomic profiling of tumor tissue and matched patient-derived cultures. **G** WES analysis of all detected mutations (w/o prediction filtering) comparing parental tumor tissue (dark orange: SCCs, dark blue: UCs) and matched cultures (light orange: p-SCCs, light blue: p-UCs), showing shared and individual mutations. **H** Oncoprint illustrating mutations predicted as “unclear” or “probably damaging” (indicated by asterisks) in key genes associated with BLCA [1, 58] and DNA repair pathways [76], stratified by subtype (orange: SCC, blue: UC) and sample origin (dark: tumor, light: cell culture). TMB (brown shades), gene alterations (grey shades), and pathway categories (green shades). Abbreviations: TMB, tumor mutational burden; BER, base excision repair; HR, homologous recombination

We established six patient-derived ex vivo cultures showing long-term growth that closely reflected the original SCC and UC tumors (passages > 10). Primary cell cultures derived from urothelial mixed tumors with partial squamous histology but lacking any evidence for keratinized markers upon ex vivo culture conditions were excluded from this study. The SCC models (p-SCC1, p-SCC2, p-SCC3) were derived from tumors with advanced invasion (pT3b); SCC1 was moderately differentiated (G2), while SCC2 and SCC3 were poorly differentiated (G3) (Suppl. Table S2). Immunohistochemistry confirmed squamous differentiation in all SCCs with strong CK5/6, p63 expression and absent GATA3, CK20 and UPK2 expression (Fig. 1; Suppl. Fig. S1). CK7 expression was negative in all SCC samples. Overall, these findings indicated high homogeneity within our SCC cohort. The corresponding cultures formed compact spheroids in 3D and sharply bordered patches in 2D.

All UC models originated from high-grade (G3) tumors, with UC1 and UC2 showing advanced invasion (pT3b and ≥ pT4a), and UC3 displaying slightly less invasion (pT3a) (Suppl. Table S2). UC3 was papillary and structured basal type and showed no signs of keratinization, similar to all other UC cases in this study. Immunohistochemical analysis revealed that UC2 showed consistent luminal differentiation, characterized by strong positivity for CK7, GATA3, CK20, and UPK2, and absence of the basal markers CK5/6 and p63 (Fig. 1; Suppl. Fig. S2). UC1 expressed CK7 (strong) and GATA3, consistent with predominant luminal type, but was negative for CK20 and UPK2. It exhibited weak p63 staining, indicating less terminal differentiation, within the luminal type constellation (GATA3+, CK5/6-) [89]. UC3, representing a basal type, expressed the basal markers CK5/6 and p63, but it lacked GATA3, CK20, and UPK2 expression, further supporting a basal molecular profile. Similar to the other UC samples, UC3 showed strong CK7 expression, clearly distinguishing the three heterogeneous UC cases from the three SCCs analyzed in this study. In culture, p-UC1 cells formed small spheroids in 3D (unsuccessful for p-UC2/p-UC3) and either spindle-shaped or patchy growth in 2D.

The tumor subtypes reflected by the patient-derived ex vivo cultures were validated by qPCR analysis of keratinization (*CNFN*, *CRNN*; Fig. 1C), basal (*KRT5*, *KRT6*; Fig. 1D), and luminal markers (*KRT7*, *GATA3*; Fig. 1E). SCC tumors and their derived p-SCC cultures consistently showed high expression of keratinization and basal markers, with minimal or absent luminal marker expression. UC1/p-UC1 and UC2/p-UC2 expressed only luminal markers (*KRT7*, *GATA3*), supporting their classification as luminal-type urothelial carcinomas, while UC3/p-UC3 expressed basal markers (*KRT5*, *KRT6*) and *KRT7* but lacked GATA3 and keratinization markers, confirming its basaloid subtype. Growth characteristics of the patient-derived ex vivo cultures (Fig. 1F) showed that p-UC1 and p-UC2 proliferated slowly (doubling times 45.1–46.0 h; corresponding to proliferation rates of 52.8–54.0% per day), whereas the p-SCC models (p-SCC1, p-SCC2, p-SCC3) grew faster (33.3–38.2 h; 67.9–77.7% per day). Notably, p-UC3 exhibited the highest proliferation rate among all models (26.2 h; 89.7% per day).

Next, we compared somatic mutation profiles of patient-derived ex vivo cell cultures and their corresponding tumors by whole-exome sequencing (WES) (Fig. 1G; Suppl. Table S3-6). WES revealed that the majority of somatic mutations were shared between tumors and their derived cultures, with overlap ranging from 57.6% (p-SCC3) to 80.5% (p-SCC2) and further underscored by copy number variation analysis (Suppl. Fig. S3A). Notably, p-UC2 displayed a lower concordance (20.4%), indicating substantial divergence. All samples displayed low TMB status (< 10 mutations/Mbp), except for UC2, which exhibited a high TMB of 12.84 mutations/Mb (Suppl. Fig. S3B). In our analyzed BLCA cohort, alterations (consensus of pathogenic prediction called “uncertain” or “probably damaging”) in known BLCA-associated genes [1, 58] were identified such as *STAG2* (33%), *CDKN2A*, *EP300 and KMT2D (*each 25%*)* an less frequent in *KRAS*, *FAT1*,* BRAF*,* ERBB2*,* KMT2B*,* ERCC2 and PIK3CA* (each 16.7%) (Fig. 1H). Most mutations were shared between tumors and their matched cell cultures, demonstrating high genomic concordance again. Notable exceptions included *KMT2B* mutations found only in tumors and *EP300* or *KMT2D* mutations restricted to cell cultures, suggesting culture-specific adaptations. Analysis of mutations in DDR/DNA repair genes revealed “probably damaging” mutations in *TP53* in all tumor tissues and ex vivo cell lines (Suppl. Fig. S3C), consistent with its well-known role in BLCA. The increase in mutant allele frequency from primary tumors (0.13–0.76) to all ex vivo cell lines (0.96–1.00) indicating clonal dominance of *TP53*-mutated cells during long-term culture. Alterations in further DNA repair genes affected various pathways (Suppl. Fig. S3D) but pathogenic variants were observed only in the HR repair and BER pathway (< 5 genes) while no mutations were detected in the specific DDR factors analyzed in this study. Furthermore, all samples were microsatellite stable (Suppl. Fig. S4,5).

### ATR inhibitor Ceralasertib radio-sensitized ex vivo cultures of SCC and UC

Novel DDR inhibitors like the ATRi Ceralasertib are currently under clinical evaluation. We, along with others, have tested the radio-sensitizing effects of ATRi in vitro, as previously demonstrated [20]. To build on these findings, we extended our analysis to patient-derived ex vivo cultures, which better recapitulate the clinical tumor environment.

**Fig. 2:**
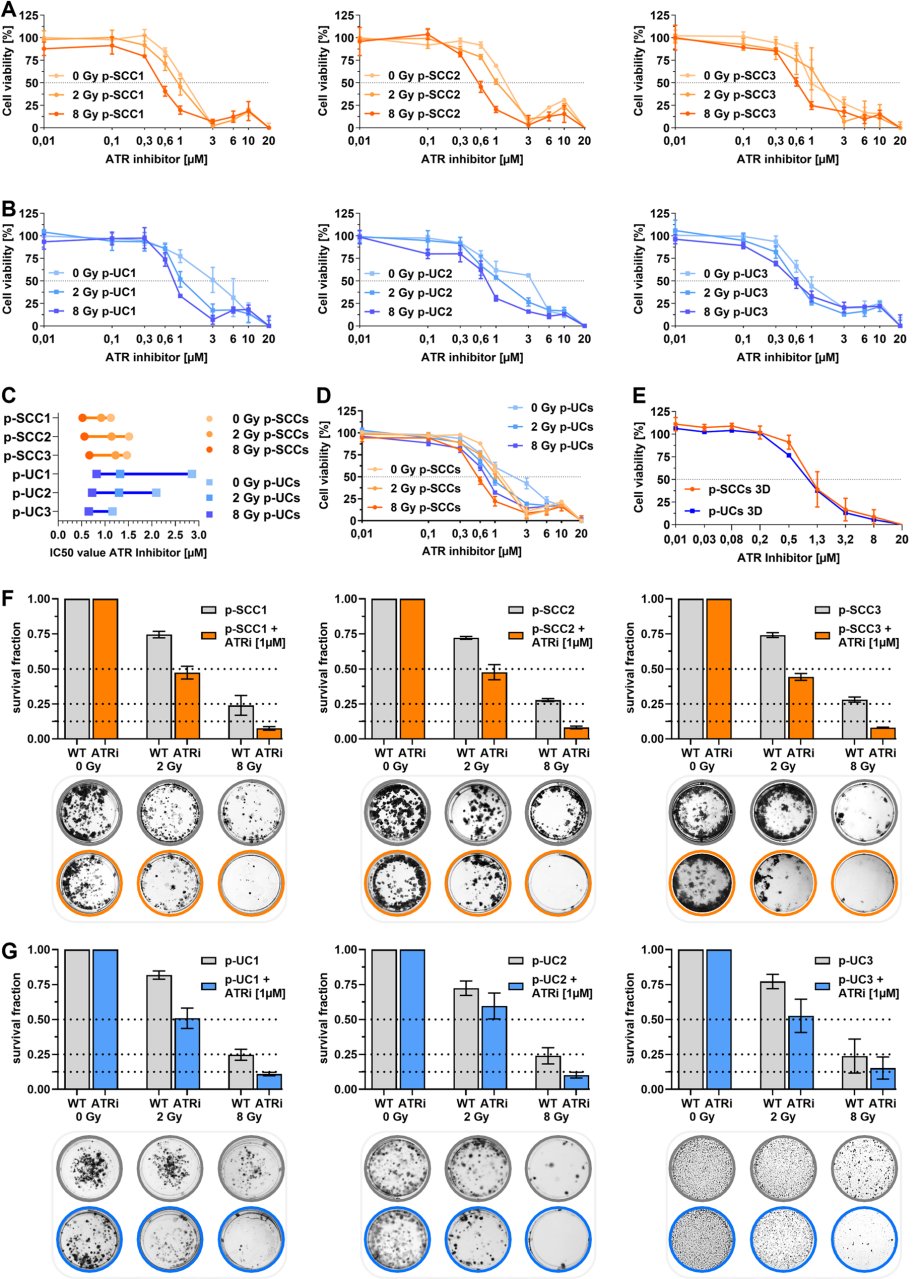
Exploring the radio-sensitizing potential of ATR inhibition in ex vivo BLCA cell models. **A-D** Short-term XTT cell viability assays were performed over 72 h to assess the effect of Ceralasertib (ATRi; 0.01–20 µM) in combination with ionizing radiation (IR; 0 Gy, 2 Gy, 8 Gy) in patient-derived 2D and 3D cultures. **A** Dose-response curves for three p-SCC cell cultures (p-SCC1, p-SCC2, p-SCC3). **B** Dose-response curves for three p-UC cell cultures (p-UC1, p-UC2, p-UC3). **C** Summary of IC50 values (µM) for all p-SCC and p-UC cell cultures across radiation doses. **D** Combined graphical representation of average dose–response behavior for all p-SCCs and p-UCs highlighting overall treatment responses. **E** Comparison of patient-derived 3D from p-SCCs and p-UCs exposed to ATRi without IR (0 Gy). **F-G** Long-term clonogenic survival assays were performed over 14 days and patient-derived 2D cultures were treated with ATRi (1 µM) and/or IR (0 Gy, 2 Gy, or 8 Gy). Top: calculated survival fractions for three p-SCC cell cultures (p-SCC1, p-SCC2, p-SCC3) or three p-UC cell cultures (p-UC1, p-UC2, p-UC3). Bottom: representative images of p-SCC or p-UC clonogenic formation

Drug-response XTT assays showed IC50 values without IR ranging from 1.11–1.51 µM in p-SCCs and 1.16–2.85 µM in p-UCs (Fig. 2A-D), with similar values in 3D cultures (p-SCC: 1.16 µM; p-UC: 0.73 µM) (Fig. 2E). Combined with IR, IC50 values decreased dose-dependently: p-SCCs from 0.92–1.22 µM at 2 Gy to 0.52–0.67 µM at 8 Gy; p-UCs from 0.65–1.32 µM at 2 Gy to 0.65–0.82 µM at 8 Gy. p-SCCs were overall more sensitive than p-UCs. Radio-sensitizing effects were also investigated for long term effects via clonogenic survival assay. Ex vivo models were treated with ATRi [1 µM] ± IR (0, 2, 8 Gy) (Fig. 2F-G). Clonogenic survival assays showed ATRi + IR significantly reduced survival. At 2 Gy, p-SCC survival fractions dropped below 0.50 (1.59-fold reduction), while p-UCs remained above 0.50 (1.42-fold reduction). At 8 Gy, survival was further reduced — 3.38-fold in p-SCCs and 2.0-fold in p-UCs. Thus, ATRi Ceralasertib effectively radio-sensitized both ex vivo cultures showing higher efficiency in cells with squamous characteristics.

### Pharmacological inhibition of ATR impeded DNA damage response after exposure to radiation

**Fig. 3:**
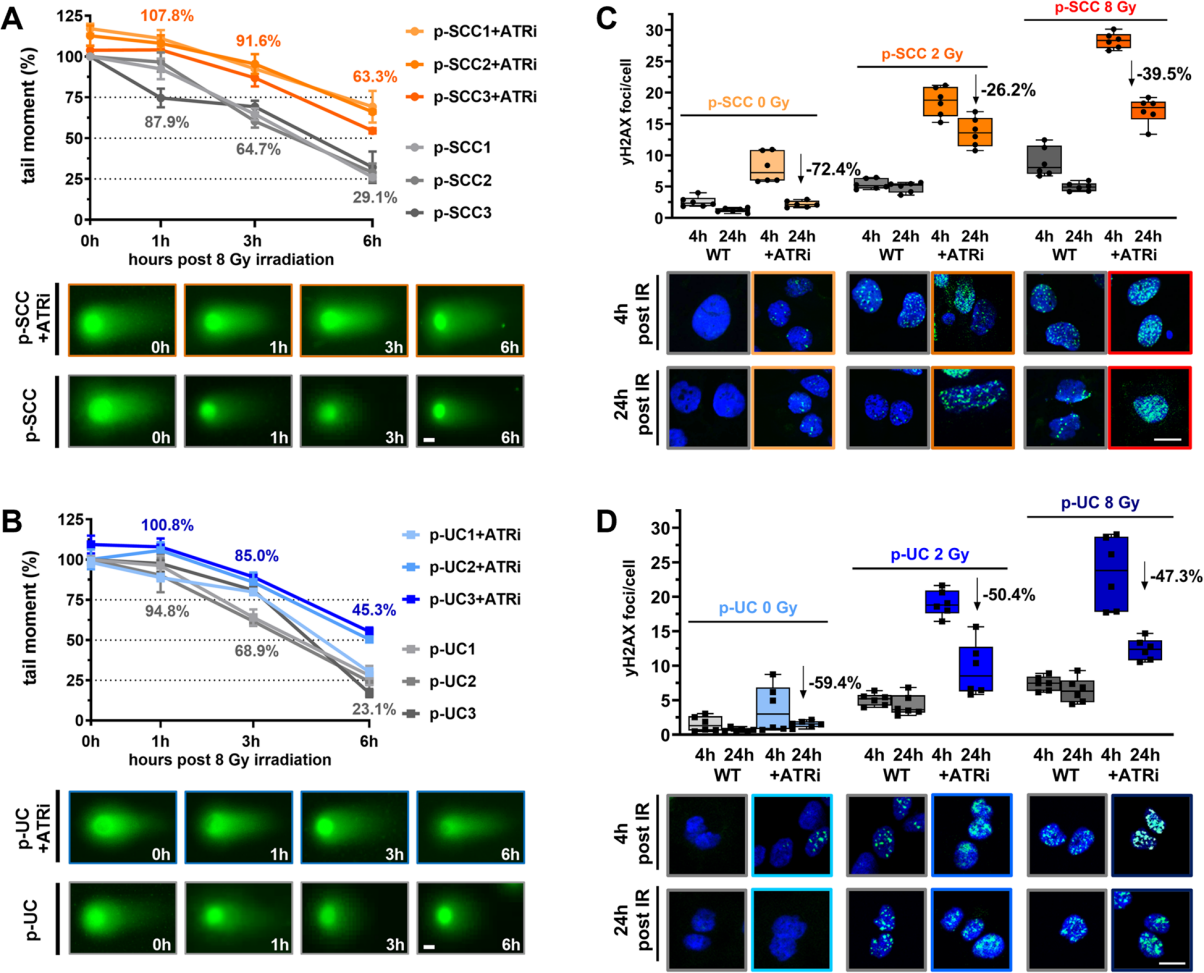
Pharmacological inhibition of ATR impeded DNA damage response after exposure to radiation. **A-B** Quantitative analysis of DNA double-strand break (DSB) repair via neutral comet assay. Patient-derived 2D cultures were exposed to 8 Gy of ionizing radiation (IR), ± ATRi (Ceralasertib, 1 µM). DNA damage was assessed by measuring tail moments at 0 h, 1 h, 3 h, and 6 h post-IR, calculated with ImageJ. At least 35 cells per condition were analyzed. **(A)** Top: tail moment for three p-SCC cell cultures (p-SCC1, p-SCC2, p-SCC3). Bottom: representative IF images of p-SCC comet tail (scale bar: 15 μm). **(B)** Same as in (**A**) for p-UC cell cultures (p-UC1, p-UC2, p-UC3). **C-D** Quantification of DNA repair through γH2AX foci analysis by IF microscopy. Patient-derived 2D cultures were exposed to 0, 2, or 8 Gy ± ATRi (Ceralasertib, 1 µM) pre-treatment followed by staining for γH2AX at 4 h and 24 h post-IR. Box plots quantify foci per nucleus calculated with ImageJ, with arrows showing a percent reduction in repair efficiency. At least 50 cells per condition were analyzed. (**C**) Top: γH2AX IF quantification as foci per cell in p-SCC. Bottom: representative IF images of γH2AX foci in p-SCC 4 h post-IR (scale bar: 20 μm). (**D**) Same as in (**C**) for p-UC cell cultures (p-UC1, p-UC2, p-UC3).

To further investigate the distinct mechanism of ATRi Ceralasertib on DNA repair, we used the neutral comet assay (Fig. 3A-B) and γH2AX IF (Fig. 3C-D). p-SCC and p-UC were treated ± ATRi applied 2 h prior to IR (8 Gy). DNA damage was assessed immediately (0 h) and at 1, 3, and 6 h post-IR. In untreated p-SCCs, tail moment (DNA fragmentation) decreased by 31–39% at 3 h and 67–74% at 6 h, indicating repair (Fig. 3A). ATRi-treated p-SCCs retained > 85% tail moment at 3 h and > 50% at 6 h, showing a 2.18-fold reduction in repair efficiency. Similarly, p-UC1 and p-UC2 showed efficient repair without ATRi, while p-UC3 had the most efficient repair (17.1% residual damage at 6 h) (Fig. 3B). ATRi delayed repair in p-UC2 and p-UC3, with tail moments above 50% at 6 h. Overall, ATR inhibition nearly doubled residual DNA damage in p-UCs (45.26% vs. 23.08% in controls).

Furthermore, γH2AX staining showed dose-dependent increases in DNA damage foci after IR. In untreated p-SCCs, γH2AX foci increased 2.1-fold (2 Gy) and 3.5-fold (8 Gy) at 4 h post-IR (Fig. 3C). ATRi treatment further amplified this to 7.2-fold and 11.0-fold, indicating unresolved damage. At 24 h, foci declined in all samples but remained elevated under ATRi, with only partial repair (reductions of 26.2% at 2 Gy and 39.5% at 8 Gy). p-UCs showed a stronger induction of γH2AX foci (1.79- and 4.79-fold without ATRi; 12.3- and 15.12-fold with ATRi at 2 and 8 Gy) (Fig. 3D). This finding confirmed results from the comet assay and highlighted subtype-specific differences in DNA repair dynamics.

### ATRi-resistant p-SCC models revealed ATR-CHK1 pathway inactivation

**Fig. 4:**
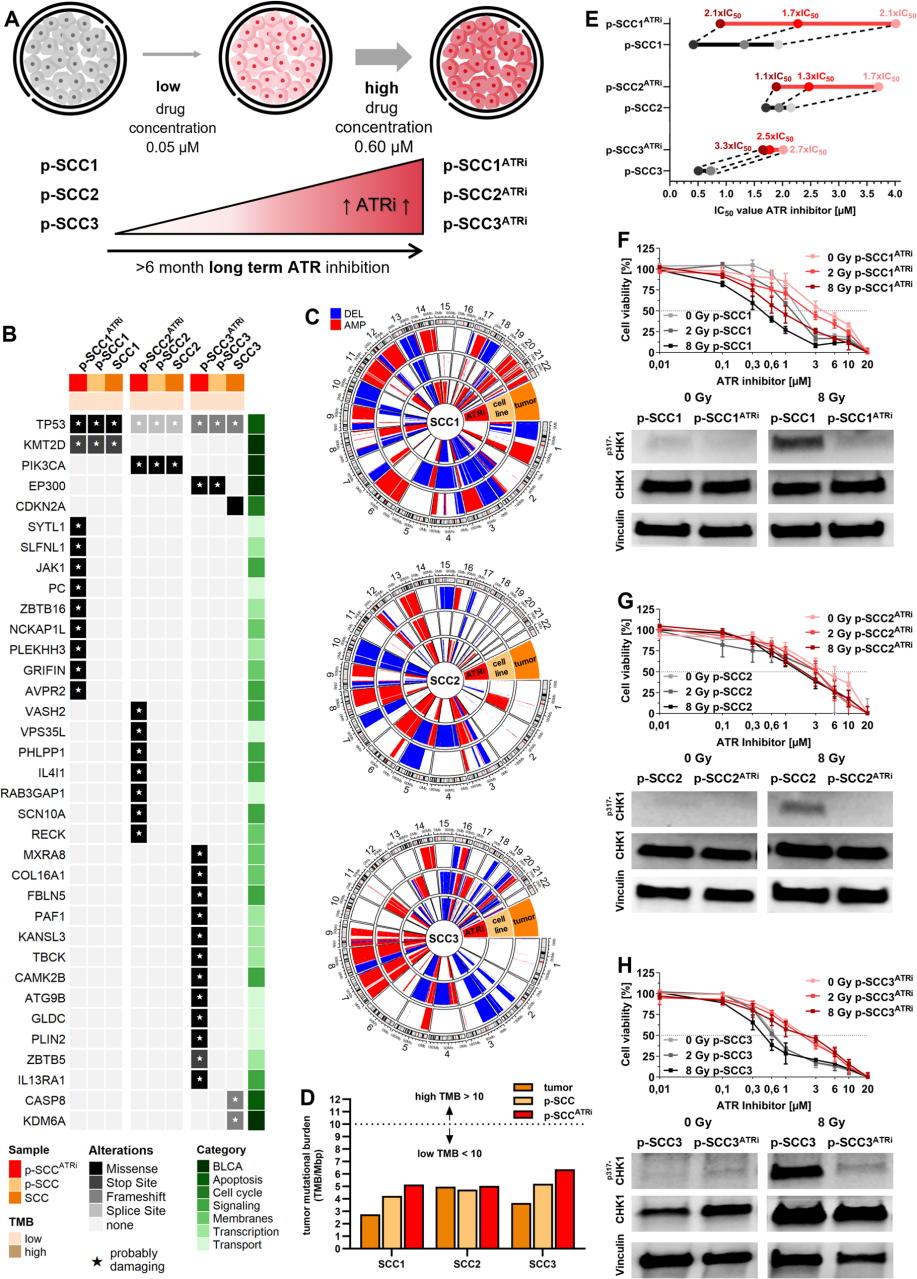
ATRi-adaptation reduced radio-sensitization and ATR–CHK1 pathway attenuation in patient-derived p-SCC models. **A** Schematic overview illustrating the generation of ATRi-adapted cell models. p-SCC1, p-SCC2, and p-SCC3 cells were continuously exposed to gradually increasing concentrations of ATRi (Ceralasertib) for over six months, resulting in adapted cell populations: p-SCC1^ATRi^, p-SCC2^ATRi^, and p-SCC3^ATRi^. **B **Oncoprint illustrating mutations predicted as “unclear” or “probably damaging” (indicated by asterisks) in key genes associated with BLCA [1, 58] and DNA repair pathways [76], as well as unique mutations identified exclusively in the resistant cell line following long-term culturing with allele frequency >0.1. Plot is stratified by sample origin (red: p-SCC^ATRi^ cell lines, light orange: parental p-SCC cell lines; dark orange: SCC tumor tissue). TMB (brown shades), gene alterations (grey shades), and pathway categories (green shades). **C** Circos plots depicting the copy number variation status of the three models across corresponding SCC tissue, parental cell line, and ATRi-resistant cell line. **D** TMB analysis revealed low status in each SCC tumor (dark orange, outer ring), the corresponding *ex vivo* cell line (light orange, middle ring), and the ATRi-resistant cell line (red, inner ring). Blue = deletion; red = amplification. **E **Summary of XTT cell viability assays showing a comparative shift in IC50 values for ATRi between parental (grey) and adapted (red) p-SCC models under 0 Gy, 2 Gy, and 8 Gy IR. Fold-changes in IC50 relative to the corresponding parental cultures are indicated, highlighting the development of resistance upon long-term ATR inhibition. **F-H **Functional validation of ATRi-adaptation in p-SCC1/p-SCC1^ATRi^, p-SCC2/p-SCC2^ATRi^, and p-SCC3/p-SCC3^ATRi^ models. Top: XTT cell viability assays (72 h) under increasing concentrations of ATRi (0.01–20 µM), ± IR (0, 2, or 8 Gy). Bottom: Immunoblot analysis of total CHK1 and phosphorylated CHK1 at Ser317. Vinculin served as loading control. Protein level analysis and raw images of immunoblots are shown in Suppl. Fig. S6 (n = 1)

To reveal compensatory effects of ATR loss due to mutational events or ATRi treatment induced resistance we used ATRi-sensitive patient-derived bladder squamous cell carcinoma cell cultures to mimic ATRi-resistant models. The use of chronic drug exposure is a valid method to develop resistance models [56, 90]. We established *n* = 3 independent ATRi-adapted ex vivo models by continuously treating the three p-SCC cell cultures with increasing concentrations (0.05–0.6 µM) of ATRi Ceralasertib over >six months (Fig. 4A**).** This stepwise treatment strategy resulted in the selection of cell populations with decreased responsiveness to ATR inhibition. The resistant, ATRi-adapted cell cultures, named p-SCC1^ATRi^, p-SCC2^ATRi^, and p-SCC3^ATRi^, remained viable under high ATRi concentrations.

WES profiling of all three ATRi-resistant cell models (Fig. 4B-D; **S**uppl. Table S3-6) did not reveal any additional pathogenic mutations within the selected BLCA–associated gene set previously analyzed in the primary tumors and their corresponding cell lines (see Fig. 1H). Notably, no new mutations were detected in the target gene *ATR*, its downstream effector *CHK1*, or any other DDR/DNA repair gene beyond those already identified in the parental tumor tissues. Consistent with these findings, all ATRi-resistant cell lines maintained a fully clonal *TP53*-mutant status (allele frequency = 1). Private mutations unique to the resistant models i.e., those acquired during long-term culturing under ATRi pressure and classified as probably damaging, affected only a small number of genes (*n* = 9 in p-SCC1^ATRi^, *n* = 7 in p-SCC2^ATRi^ and *n* = 12 in p-SCC3^ATRi^) without any observed enrichment of distinct pathways or overlaps between resistant models. Furthermore, all acquired mutations exhibited allele frequencies < 0.32, indicating their presence only in subclones of the resistant cell lines. The only exception was one mutation in *PLEKHH3*, which displayed a notably higher allele frequency of 0.77. The median CNV status (2) was similar across all resistant models and compared to their parental line (Fig. 4C). The TMB scores of the resistant cell lines were slightly increased but still remained at lower levels (< 10 mutations/Mbp) when compared to that of their respective parental cell lines and tumor tissue (Fig. 4D).

To validate ATR pathway inactivation in the p-SCC^ATRi^ models, we performed XTT cell proliferation assays (Fig. 4E-H, Suppl. Fig. S6) and assessed downstream signaling by CHK1 protein levels. XTT assays showed that all p-SCC^ATRi^ models exhibited a consistent shift toward higher IC50 values compared to their untreated control. This effect was modest in p-SCC2^ATRi^ (0 Gy: 1.1-fold, 2 Gy: 1.3-fold, 8 Gy: 1.7-fold), more pronounced in p-SCC1^ATRi^ (0 Gy: 2.1-fold, 2 Gy: 1.7-fold, 8 Gy: 2.1-fold), and strongest in p-SCC3^ATRi^ (0 Gy: 3.3-fold, 2 Gy: 2.5-fold, 8 Gy: 2.7-fold), indicating varying degrees of acquired resistance to ATR inhibition across the models. To confirm functional dysregulation of ATR driven downstream signaling, we investigated CHK1 activation, a direct and main downstream target of ATR, by evaluating its phosphorylation at Ser317 via immunoblot. We observed a blocked ATR–CHK1 signaling axis, evidenced by decreased levels of pSer317-CHK1 upon radiation induced DNA damage, while total CHK1 protein levels were increased.

### ATRi-resistance caused transcriptomic and kinetic shift towards compensatory mechanisms for genomic stability

**Fig. 5:**
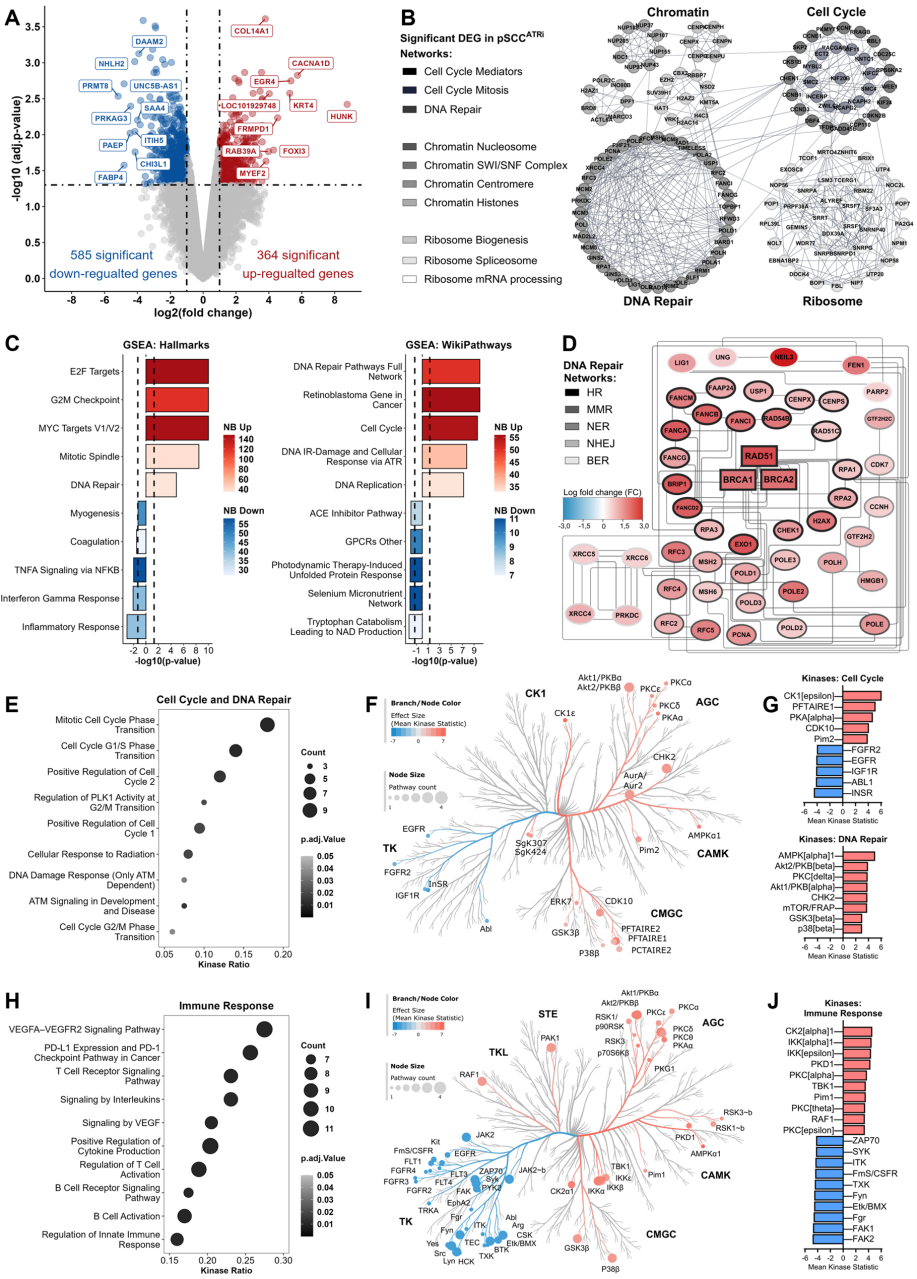
Transcriptomic analysis revealed compensatory mechanisms in ATRi-resistant p-SCC models. **A** RNA-Seq Volcano plot showing differentially expressed genes (DEGs, |FC| ≥ 2.0 and p-value ≤ 0.05) in p-SCC1/2/3^ATRi^versus parental p-SCC1/2/3 models. Red: upregulated; blue: downregulated; top 10 DEGs are labeled. **B **Functional interaction network of DEGs enriched in cellular pathways. Nodes represent individual genes; edges indicate known/predicted interactions. Red: upregulated; blue: downregulated. **C **RNA-Seq GSEA bar plots showing pathway enrichment scores from Hallmark and WikiPathways databases. Further GSEA and GSVA analyses are provided by Suppl. Fig. S7 and Suppl. Fig. S8. **D **Heatmap of orthogonal clustering of DEGs from *WikiPathways DNA Repair Pathways Full Network* (WP4663), illustrating transcriptional reprogramming in DNA repair pathways. **E**,** H **Kinase activity dot plot illustrating enrichment of processes related to cell cycle and DNA repair (**E**), and immune response (**H**) according to represented kinase ratio. Dot size reflects number of associated kinases; color gradient indicates statistical significance (adj. p-value). **F**,** I **Kinase coral tree network diagrams visualizing interactions in cell cycle (**F**) and immune signaling (**I**). Node size represents mean rank; node color indicates effect size (red = activated, blue = inactivated); edges denote functional associations. Kinases are grouped by family: CK1 (Casein Kinase 1), AGC (Protein Kinase A, G, and C family), CAMK (Calcium/Calmodulin-dependent Protein Kinase), CMGC (CDK, MAPK, GSK3, CLK family), TK (Tyrosine Kinase), TKL (Tyrosine Kinase-Like), STE (Homologs of yeast Sterile kinases). **G**,** J **Bar plots summarizing mean rank differences of key kinases involved in cell cycle and DNA repair (**G**) and immune response (**J**). Bars are color-coded by direction of regulation (Red: activated; blue: inactivated), highlighting kinases with significant shifts (Mean Kinase Statistic ≥ 3.0, adj. p-value ≤ 0.05) in activity in p-SCC^ATRi^ models.

To uncover mechanisms enabling p-SCC^ATRi^ models to maintain genomic stability despite ATRi resistance, we performed transcriptomic and kinome profiling (Fig. 5; Suppl. Table S7-9). Differential gene expression (DEG) analysis revealed 585 downregulated and 364 upregulated genes (Fig. 5A). Among the most upregulated genes, we identified *COL14A1* and *KRT4* linked to extracellular matrix remodeling and keratinization [91, 92]. *ITIH5*, a putative tumor and metastasis suppressor gene [93], known to affect epigenetic reprogramming [94] that has been described to affect therapy response in basal-squamous-like BLCAs [95] was shown to be strongly downregulated. Pathway enrichment (Fig. 5B) of the significant DEG sets (FC >1.5, p-value < 0.05) indicated broad transcriptional reprogramming beyond canonical ATR signaling [26] impacting chromatin organization (e.g., nucleosome assembly, SWI/SNF complex, histone regulation) and nucleolar functions (e.g., mRNA synthesis, ribosomal biogenesis, spliceosome), highlighting ATR’s multifaceted role.

Gene Set Enrichment Analysis (GSEA) revealed enrichment of DNA repair pathways, including Hallmark *DNA Repair* (M5938), WikiPathways *DNA Repair Pathways Full Network* (WP4663), and KEGG pathways for *Nucleotide Excision Repair* (hsa03420), *Homologous Recombination* (hsa03440), *Mismatch Repair* (hsa03430), and *Base Excision Repair* (hsa03410) (Fig. 5C, Suppl. Fig. S7). Orthogonal clustering of genes within these gene sets confirmed predominant upregulation of HR-related genes (Fig. 5D), particularly *RAD51*, *BRCA1*, and *BRCA2*. Cell cycle–related gene sets (*WikiPathways Cell Cycle* (WP179), *KEGG Cell Cycle* (hsa04110), *E2F Targets* (M5925), *G2/M Checkpoint* (M5901), *MYC Targets* (M5928), and *Mitotic Spindle* (M5895)) were also enriched, indicating increased proliferative capacity under ATRi. In contrast, immune response pathways (e.g. *Inflammatory Response* (M5932), *Interferon-γ Response* (M5913), and *TNFα Signaling via NF-κB* (M5890)) were significantly downregulated. GSEA results were validated by Gene Set Variation Analysis (GSVA), particularly confirming the upregulation of cell cycle regulation and DNA repair signaling pathways (Suppl. Fig. S8).

To further explore the regulatory landscape upon DDR activity, we performed kinase activity screening based on transcriptomic signatures post-IR (Fig. 5E–J; Suppl. Table S10). Kinase enrichment revealed upregulation of cell cycle–associated pathways, including *Mitosis* (GO:0044772), *G1/S Transition* (GO:0044843), *G2/M Checkpoint Control* (R-HSA-2565942), and *DNA Damage Response* (WP710) (Fig. 5E). Key kinases such as CK1 [96], CDK10 [97], CAMKs [98], and AKT1/2 showed increased activity (Fig. 5F, G), supporting checkpoint control and HR repair via BRCA1/RAD51 recruitment [99]. In contrast, immune-related kinases (ZAP70 [100], SYK [101], ITK [102], and CSF1R [103]) displayed reduced activity, implicating dampened immune signaling in pathways like *PD-L1 Expression and PD-1 Checkpoint in Cancer* (hsa05235), *T Cell Receptor Signaling* (hsa04660), and *B Cell Receptor Signaling* (WP23) (Fig. 5H). In contrast, pro-survival kinases (CK2 [104], IKKs [105], RAF1 [106]) were also activated (Fig. 5I, J) promoting proliferation and anti-apoptotic signaling in p-SCC^ATRi^ cells.

### ATRi-resistance caused an epigenetic shift as putative molecular memory to modulate DNA damage response including HR

**Fig. 6:**
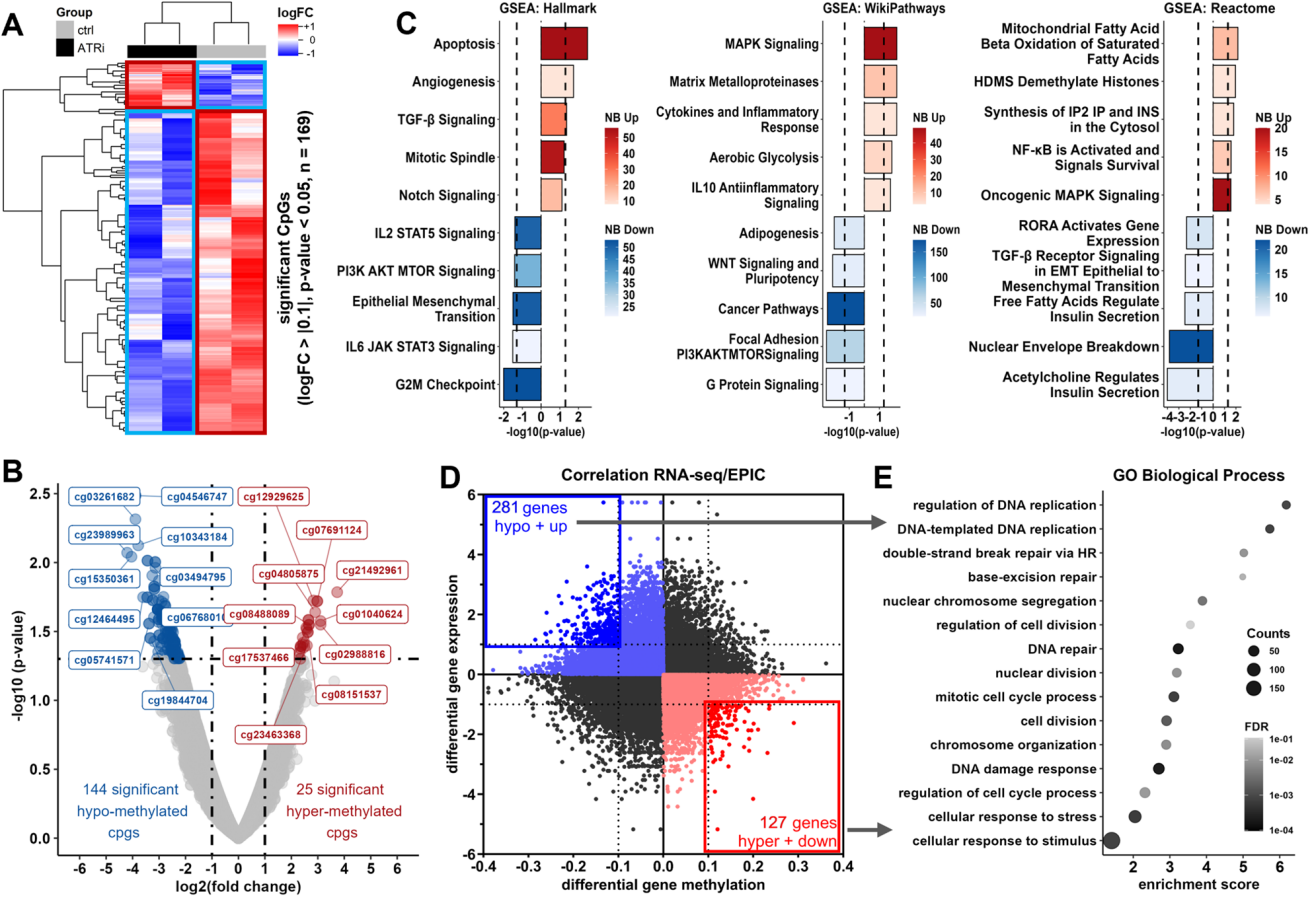
DNA methylation shift in ATRi-resistant models as putative memory for compensatory pathway regulation. **A** Illustration of a DNA methylation shift. A heatmap of significantly differentially methylated CpGs (|logFC| ≥ 0.1 and p-value ≤ 0.05) in p-SCC1/3^ATRi^ versus parental p-SCC1/3 models from EPIC-Array dataset is shown, clustered by Spearman correlation. Red: hypermethylated; blue: hypomethylated. **B** Volcano plot of CpGs in (**A**) showing differentially methylated CpGs; the top 10 CpGs are labeled. **C** Gene set enrichment analysis (GSEA) based on unsupervised EPIC-Array dataset showing enrichment scores on the gene level for pathways from Hallmark, WikiPathways, and Reactome databases. **D** Correlation analysis of transcriptomic and methylomic changes in ATRi-resistant models. Quadrant chart displays differential methylation (x-axis) versus differential expression (y-axis). Light blue: hypomethylated with upregulated expression; light red: hypermethylated with downregulated expression; dark blue: hypomethylated (logFC < − 0.1) with strongly upregulated expression (logFC > + 1); dark red: hypermethylated (logFC > + 0.1) with strongly downregulated expression (logFC < − 1). **E **Dot plot showing GO biological process enrichment based on the correlated dataset of (**D**). Biological process terms (y-axis) are displayed with enrichment scores (x-axis) and FDR (dot size, < 0.05).

Since epigenetic mechanisms are known to anchor transcriptomic networks driving drug resistance [107], we focused on genome-wide DNA methylation. To this end, methylome analyses were performed using Infinium MethylationEPIC BeadChip arrays across ATRi-resistant models compared to long-term growth DMSO controls (Fig. 6; Suppl. Table S11). These analyses revealed a pronounced epigenetic shift as cluster analyses of differentially methylated CpGs confirmed a clear separation of resistant from parental-like control lines, indicating a convergent, lineage-independent methylation signature (Fig. 6A). Furthermore, across the independent ATRi-resistant models, we identified significantly hypo- and hypermethylated CpG sites. In fact, 144 significantly hypomethylated (logFC >0.1; *p* *<* 0.05) and 25 hypermethylated (logFC < − 0.1; *p* < 0.05) CpGs were revealed (Fig. 6B). Beyond that, GSEA highlighted pathway-specific alterations (Fig. 6C). CpG sites showing hypermethylation were mainly enriched in gene sets related to processes like Hallmark *Apoptosis* (M5902), *Mitotic Spindle* (M5893), and WikiPathways *Cytokines and Inflammatory Response* (M39711). In contrast, CpGs mapping to cell cycle–associated pathways such as *G2M Checkpoint* (M5901) and immune-activating signatures like *IL-2/STAT5* (M5947) or *IL-6/JAK/STAT3 Signaling* (M5897) showed hypomethylation.

These findings supported our notion that DNA methylation could act as molecular memory in ATRi-resistant models, regulating downstream consequences and enabling pathway bypass mechanisms. Integration of individual RNA-Seq data with CpG methylation profiles on gene level from each ATRi-resistant model revealed an inverse correlation for 408 genes (Fig. 6D; Suppl. Table S12). Specifically, 127 genes displayed hypomethylation (logFC < − 0.1) accompanied by increased expression (FC > 1), potentially indicating gene activation (highlighted red), whereas 281 hypermethylated genes (logFC > 0.1) showed concomitant downregulation (logFC < − 1), suggesting gene inactivation (highlighted blue). Gene Ontology (GO) enrichment analysis of these candidates highlighted processes related to DNA repair and cell cycle regulation, including double-strand break repair via homologous recombination (GO:0000724), base-excision repair (GO:0006284), and mitotic cell cycle process (GO:1903047) (Fig. 6E; Suppl. Table S13). Notably, these pathways involved DNA repair genes such as *POLQ*, *RPA2*, and *XRCC4*, and HR-specific genes including *BRCA1*, *RPA2*, and members of the MCM complex (*MCM2*/*4*/*5*/*7*), highlighting again the central role of DNA damage response and replication control in ATRi-resistance.

### Targeting RAD51 bypassed ATRi-resistance and promoted DNA damage and cell death

**Fig. 7:**
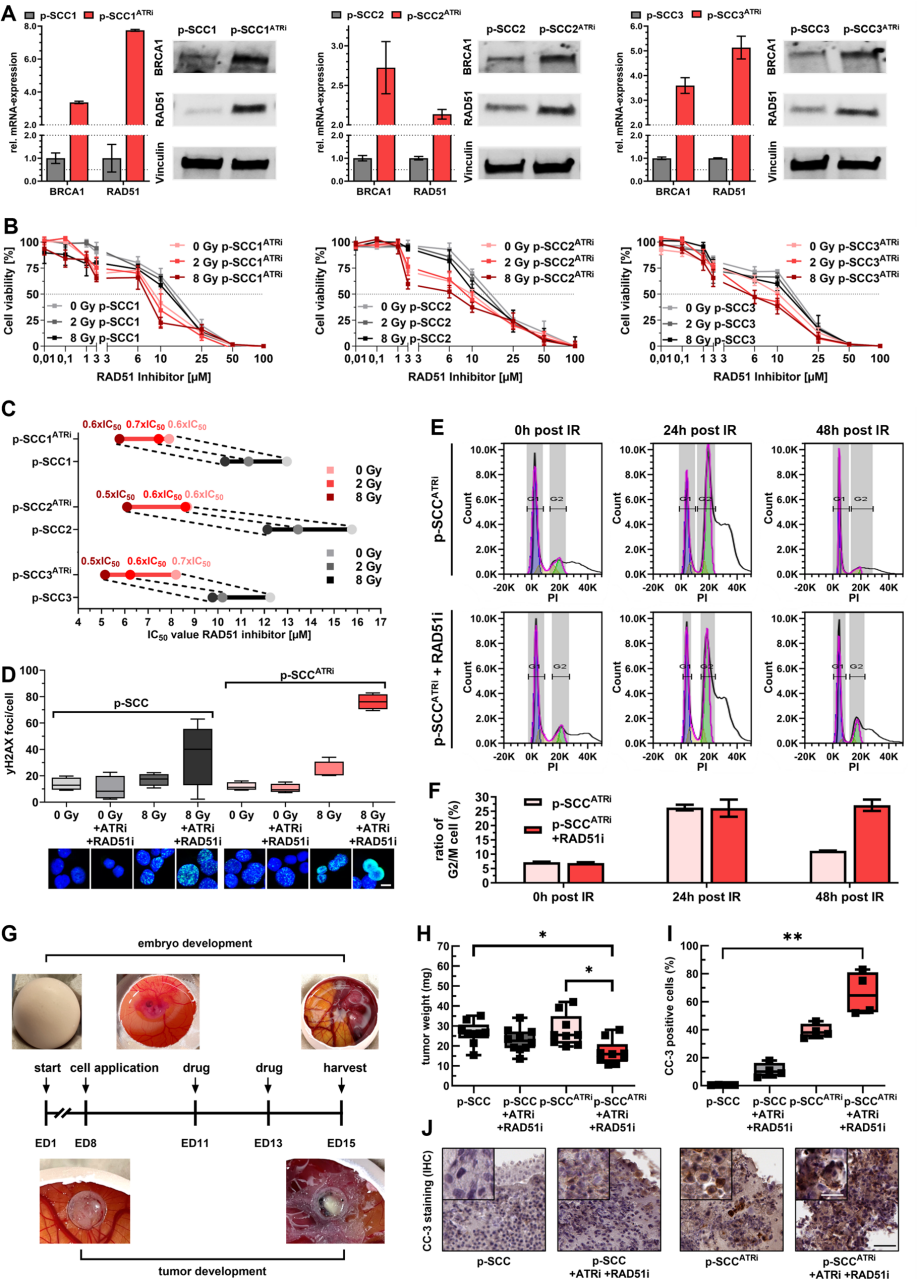
Homologous recombination repair dependency as an acquired vulnerability in ATRi-resistant p-SCC models. **A** Expression of key HR repair genes in controls (grey) vs. p-SCC^ATRi^ cells (red). Left: relative *BRCA1* and *RAD51*-mRNA expression (normalized to *GAPDH*). Right: corresponding immunoblots for BRCA1 and RAD51 (normalized to Vinculin). Protein level analysis and raw blots are shown in Suppl. Fig. S9 (n = 1). **B** XTT cell viability assays evaluating sensitivity of controls (grey) and p-SCC^ATRi^ cells (red) to RAD51 inhibitor (RAD51i, B02, 0.01–100 µM), alone or with IR (0, 2, 8 Gy). **C **IC50 summary for RAD51i derived from (**B**). IC50 Fold-decreases in p-SCC^ATRi^ cells (red) relative to controls (grey) are shown across radiation doses (0, 2, 8 Gy). **D** DNA repair quantified via γH2AX foci (IF microscopy) in controls(grey) and p-SCC^ATRi^ cells (red) treated with DMSO (ctrl) or ATRi (1 µM) with RAD51i (5 µM) ± 8 Gy IR. ≥ 50 cells per condition were analyzed; representative images are shown (scale bar: 20 µm). **E**-**F** Cell cycle analysis by flow cytometry. **E** FACS histograms at 0, 24, and 48 h post-IR in untreated(top) and combined ATRi and RAD51i treated p-SCC^ATRi^ cells (bottom), ≥ 10,000 cells were analyzed. **F** Quantification of G2/M phase percentages in untreated (light red) and ATRi + RAD51i treated p-SCC^ATRi^ cells (red). **G-I** *In ovo* chicken chorioallantoic membrane (CAM) xenograft model with p-SCC and p-SCC^ATRi^ cells. **G** Setup: cells were implanted on embryonic day (ED) 8, and treated with vehicle, ATRi (1 µM), or combination with RAD51i (5 µM) at ED11 and ED13. Tumors were harvested at ED15. **H** Xenograft weights (p-SCC n = 9; p-SCC^ATRi^ n= 9–10). **I** Apoptosis quantified by cleaved caspase-3 (CC-3) staining in formalin-fixed xenografts (four regions/sample, pathologist assessment; n = 4). **J** Representative CC-3 IHC images from xenografts (black scale bar: 50 µm, white scale bar: 20 µm).

In order to validate the functional relevance of revealed compensations due to ATRi-resistance with druggable potential, we confirmed upregulation of DNA repair genes in p-SCC^ATRi^. qPCR analysis of HR repair factors revealed 2.7–3.6-fold increase in *BRCA1* and a 2.1–7.7-fold increase in *RAD51* expression in p-SCC^ATRi^ models versus controls (Fig. 7A). These findings were validated at the protein level, showing 1.7–2.6-fold higher BRCA1 and 1.8–6.1-fold higher RAD51 expression (Suppl. Fig. S9A-C). In addition, NHEJ factors were affected, as demonstrated by a 5.7-fold increase in DNA-PK activation (Ser2056 phosphorylation) and a moderate 1.7-fold increase in Artemis activation (Ser516 phosphorylation) in p-SCC^ATRi^ models (Suppl. Fig. S9D-F). These results indicated that ATR inactivation impaired double-strand break repair, leading to compensatory activation of both HR repair and NHEJ pathways, with HR and particularly RAD51 showing the strongest upregulation.

To address whether the observed DNA repair compensations were due to direct relationships between ATR and HR rather than from adaptive, nonspecific responses over months due to chronic drug pressure, we performed two complementary experiments in wild-type p-SCC1 cells (Suppl. Fig. S10). First, we generated a transient *ATR* knockdown model by transfecting wild-type cells with *ATR*-specific siRNA. This resulted in a transient 0.54-fold reduction in *ATR*-mRNA expression, consistent with decreased ATR protein levels 96 h post-transfection (Suppl. Fig. S10A). *ATR* downregulation was accompanied by a 2.1-fold increase in *RAD51* mRNA expression in line with elevated RAD51 protein levels 144 h post-transfection (Suppl. Fig. S10B). Second, we applied short-term pharmacologic ATR inhibition with Ceralasertib (1 µM for 24–96 h) in wild-type cells (Suppl. Fig. S10C). Here, we observed a 3.8-fold increase in *RAD51* mRNA expression and corresponding protein levels 96 h post-inhibition. In addition, *BRCA1* mRNA was also upregulated by 2.5-fold in line with elevated BRCA1 protein level after 96 h. In untreated control cells, RAD51 and BRCA1 levels remained stable. Together, these data demonstrated, although less pronounced than in our ATRi-resistant models, that ATR loss triggered upregulation of HR repair factors within a short-term period indicating a direct feedback response.

Given this dependency, we finally aimed to target HR repair using the RAD51 inhibitor (RAD51i) B02. XTT assays revealed increased sensitivity of p-SCC^ATRi^ models to RAD51i (Fig. 7B, C), with IC50 reductions of 34–44% in p-SCC1^ATRi^ and > 50% in p-SCC2^ATRi^ after 8 Gy IR. p-SCC3^ATRi^ showed consistent IC50 shifts (30–47%), highlighting HR repair dependency in all ATRi-adapted cells. Furthermore, RAD51i impaired DNA repair more strongly in p-SCC^ATRi^ than in controls, as γH2AX staining at 24 h post 8 Gy IR showed elevated DNA damage (76 foci/cell in p-SCC^ATRi^+ RAD51i vs. 35 foci/cell in controls) (Fig. 7D). Next, cell cycle analysis showed similar G2/M populations for p-SCC^ATRi^ and p-SCC^ATRi^+ RAD51i at 24 h (26.3% vs. 26.1%). However, after 48 h, p-SCC^ATRi^+ RAD51i cells maintained G2/M arrest (27.1%), whereas the control dropped to 11.2% (Fig. 7E, F). These results confirmed that HR repair inhibition in p-SCC^ATRi^ induced prolonged cell cycle arrest.

To finally assess treatment efficacy of RAD51i due to ATRi-dysfunction, we used the chorioallantoic membrane (CAM) *in ovo* model, with the experimental timeline outlined in Fig. 7G. Tumor weights of p-SCC^ATRi^+ RAD51i xenografts were significantly lower (16.8 mg) compared to p-SCC (26.5 mg) and p-SCC^ATRi^ (22.8 mg), and a similar trend was observed in the p-SCC + ATRi + RAD51i with 27.8 mg (Fig. 7H). This tumor growth inhibition correlated with enhanced apoptotic activity, as indicated by cleaved caspase-3 (CC-3) immunostaining. While p-SCC controls showed ~ 15% CC-3 positive cells, p-SCC^ATRi^ xenografts had 39%, and p-SCC^ATRi^+ RAD51i reached 66% positivity (Fig. 7I, J), confirming apoptosis as the main mechanism of tumor suppression.

### Therapeutic strategies for ATRi or RAD51i application based on genetic profile of BLCA patients

**Fig. 8:**
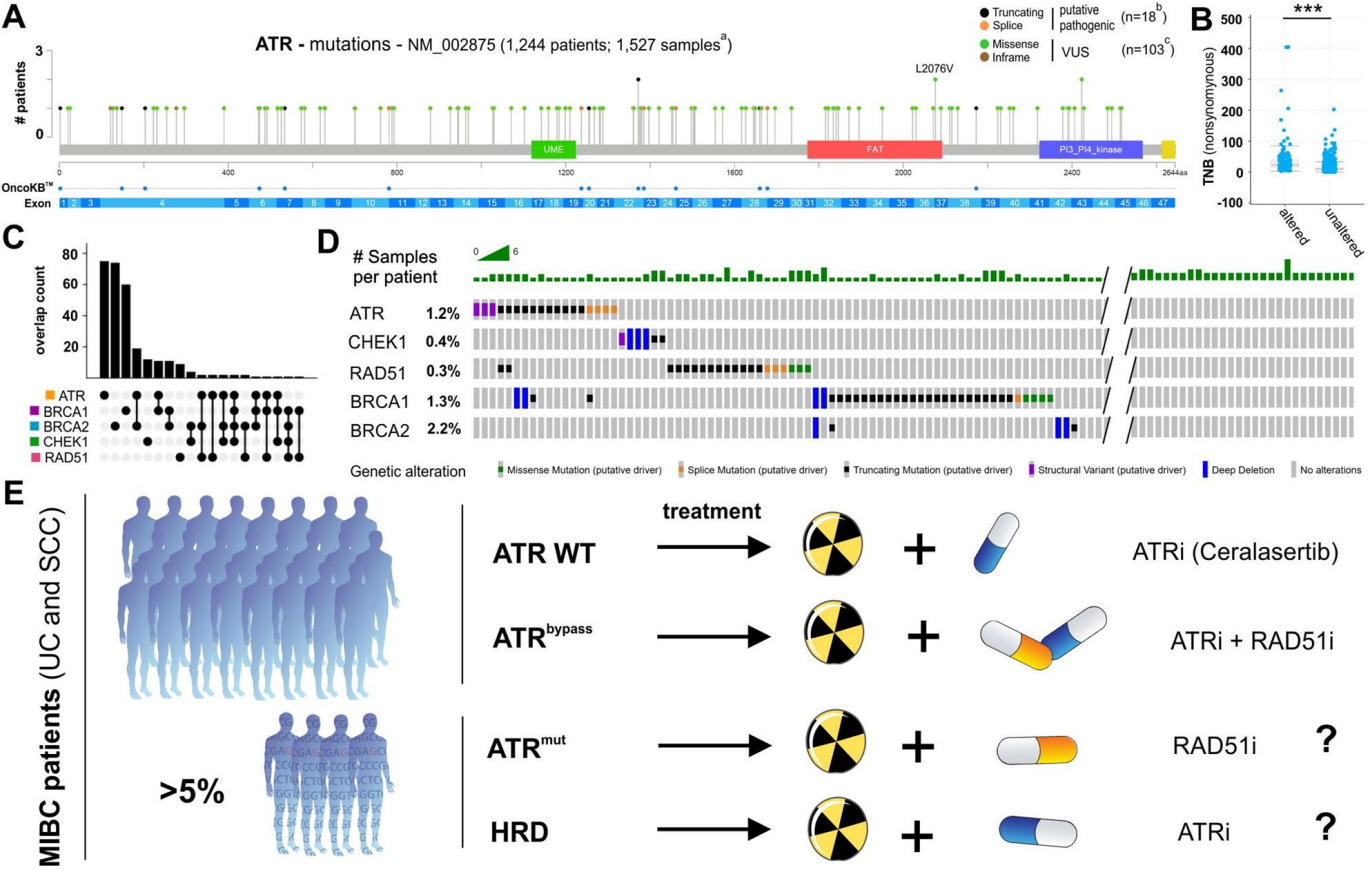
Genetic profile–guided therapeutic approaches for ATR or RAD51 inhibitor treatment in BLCA. **A** Lollipop with distribution of *ATR* mutations (single nucleotide variants (SNV) comprising pathogenic mutations and variants of uncertain significance (VUS)), as observed in 1,527 samples (1,244 patients). Samples that had not been profiled for all genes of the following analyses in analyzed profiles were excluded from analysis (*n* = 132). UME domain (green, upstream regulatory region), FAT domain (red, FRAP–ATM–TRRAP), PI3/PI4 kinase catalytic domain (blue) and FATC domain (yellow). The presented number of ATR mutations (^a^ overall 1,527; ^b^putative pathogenic 18; ^c^VUS 103) was adjusted by excluding duplicate mutations of patients with multiple samples (overall *n* = 19; i.e. *n* = 4 pathogenic and *n* = 15 VUS). **B** Wilcoxon test demonstrated a close link between TMB status and patients with genetic *ATR* alterations (altered) compared to those samples lacking mutations (unaltered) (p-value < 10^− 10^). **C** Intersection plot illustrating co-occurring mutations of *ATR* (orange), *BRCA1* (blue), *BRCA2* (pink), *CHEK1* (green), and *RAD51* (purple) in TCGA BLCA patient cohort. **D** Oncoprint showing only potentially pathogenic mutations of those five genes in all analyzed samples of the TCGA BLCA cohort (excluding *n* = 132 samples without information for all analyzed genes). Mutation types: *missense* (green), splice site (orange), truncating (black), structural variants (purple), deep deletions (blue), no alteration (grey). **E** Cartoon with hypothetical treatment strategies related to genetic alterations for ATR and RAD51 inhibitors. ATR wild-type (ATR WT): treatment with ATRi combined with ionizing radiation. ATR^bypass^ (ATRi-resistant with HR repair activation): dual inhibition using ATRi and RAD51i. ATR-mutated (ATR^mut^): RAD51 inhibition. HRD (HR-deficient tumors): potential sensitivity to DNA damage response inhibitors. Overall frequency of patients harboring either ATR^mut^ or HRD was 5.6% (70 out of 1,244 patients).

Considering the experimental data suggesting a potential dependency of ATR-deficient tumors on HR, we finally analyzed the genomic landscape of both DDR pathways using the TCGA BLCA cohort independently of a given subtype (Fig. 8). Overall, the *ATR* gene, located on chromosome 3q23, consisting of 47 exons, was genetically altered in 7.9% of BLCA samples (121 out of 1,527 after excluding 19 duplicate mutations in patients with multiple samples). Positions of single nucleotide variants (SNVs) across the gene are shown in Fig. 8A. This “altered” group comprised 18 mutations with a potential pathogenic impact, such as truncating mutations, and 103 variants of uncertain significance (VUS). When comparing altered and unaltered samples, ATR genetic variants were significantly associated with a higher TMB status (Fig. 8B). To assess the co-occurrence of mutations in both DNA repair pathways (*ATR-CHEK1* and HR), we analyzed the overlap of genetic alterations in five key genes across the samples: *ATR*, *BRCA1*, *BRCA2*, *CHEK1*, and *RAD51* (Fig. 8C). Most of the mutated BLCA samples showed exclusive alterations only in a single gene (79.3%; 230 out of 390 altered samples). Comparing *ATR-CHEK1* and HR, we identified a total of 87 samples with altered *ATR-CHEK1* genes, and 157 samples associated with the HR repair pathway while excluding samples with alterations in the other DDR pathways. Regarding pathogenic mutations, we still observed 1.6% (24 out of 1,527 samples) with a putative impaired *ATR-CHEK1*-repair axis and 3.8% (59 out of 1,527 samples) with a putative HR-deficiency (HRD). An overlap of mutations potentially affecting both DDR pathways was detected in only 0.4% of all samples (Fig. 8D).

Since we demonstrated that RAD51 inhibition effectively targets the HR repair pathway in ATRi-adapted p-SCC models, we propose multifaceted therapeutic concepts particularly when considering patients with distinct genetic backgrounds in one of the two DDR signaling pathways (ATR or HR) (Fig. 8E). In this context, more than 5% of patients diagnosed with bladder carcinomas may be eligible for at least one of the proposed therapeutic approaches.

## Discussion

ATR is a major kinase in the DDR pathway and a central regulator of genomic integrity [26, 108]. Its essential role in maintaining genome stability, particularly during replication stress, makes ATR an attractive target for cancer therapy [22, 25]. Preclinical studies and early-phase clinical trials have already demonstrated the antitumor effects of ATRis, which are generally well tolerated due to the tumor-selective pressure imposed by pre-existing replication stress [27, 28, 33–36, 109–112].

Building upon our previous findings using the ATRi Ceralasertib in the SCC cell line SCaBER [20], we now extended our analysis to patient-derived ex vivo models established from cystectomy specimens of patients diagnosed with UC or the rare SCC. We are aware that the number of patient-derived cultures in this study is limited. However, since we were particularly interested in establishing valid SCC research models, the rarity of this subtype (2–5% of bladder tumors [113]) limited access to suitable biological material. This is in line with previous studies involving urinary SCC [14, 114] and represents one of the main challenges in clinical trials, with the consequence of a substantial gap in evidence-based recommendations for novel therapies in SCC. However, our patient-derived ex vivo SCC models had to meet distinct criteria to ensure a homogeneous and valid model reflecting squamous bladder cancer. In fact, p-SCCs and p-UCs retained the key histological, genetic, and molecular hallmarks of the respective tumor types and lacked mutations in our predefined target genes, thereby providing a reliable platform for the functional assessment of targeted therapies. Moreover, our models harbored common BLCA pathogenic gene variants such as *KMT2D*, *PIK3CA*, *EP300*, and *TP53* [1], supporting their relevance for translational research.

Our results confirmed strong radio-sensitizing effect of Ceralasertib in SCC models at clinically relevant concentrations. In combination with IR, ATR inhibition resulted in decreased IC50 values, reduced clonogenic survival, and increased DNA damage signaling. Although UC models also exhibited responses to Ceralasertib, the effect was significantly more pronounced in p-SCCs. This is particularly noteworthy, considering the limited therapeutic options currently available for this rare and aggressive BLCA subtype [1, 2]. Mechanistically, our data support that ATR inhibition exploits a synthetic-lethal interaction in tumors with p53 loss, a common event in BLCA [1, 115, 116]. Without p53, cells fail to arrest at G1/S checkpoint despite DNA damage and become dependent on ATR to maintain S and G2/M checkpoints. ATR inhibition disrupts this control, leading to replication stress, mitotic catastrophe, and cell death [22, 26, 108]. While our focus was on bladder SCC, these findings likely extend to other squamous cancers such as those of the head and neck, esophagus, and cervix, where high replication stress and frequent TP53 mutations are also common.

When considering novel therapeutic strategies, it is crucial to understand not only the primary function of the target but also the broader downstream consequences of its inhibition to anticipate resistance mechanisms and guide combination approaches. Our multi-dimensional profiling of ATRi-resistant SCC models revealed that ATR’s molecular background extends beyond DDR to influence nuclear structure and gene regulation. We observed changes in chromatin remodeling (e.g., SWI/SNF complex), nucleosome assembly, histone regulation, and nucleolar functions. Notably, studies in yeast showed that the SWI/SNF complex can activate the ATR ortholog Mec1, indicating a non-canonical epigenetic role for ATR beyond DNA repair [117]. While our data suggest a role for chromatin remodeling in ATRi-resistance, functional validation of SWI/SNF dependency will be essential to strengthen this conclusion, particularly in light of the previously reported synergism between ATR and SWI/SNF complex inhibition [118] and must be addressed in future studies. Thus, ATR inhibition may elicit broader cellular consequences than previously assumed, extending to epigenetic regulation and nuclear homeostasis, which likely contribute to the transcriptomic and kinomic alterations we observed. This suggests that ATR inhibition affects epigenetic regulation and nuclear homeostasis, consistent with findings by Kidiyoor et al. (2016, 2020) who demonstrated ATR’s involvement in maintaining chromatin integrity and nuclear architecture independently of DDR [26, 108]. Thus, ATR may act as a multifaceted regulator in BLCA, influencing genome stability, as well as transcriptional and epigenetic programs.

Moreover, ATR inhibition has been increasingly linked to modulation of the immune response, as reviewed by Mavroeidi et al. (2024) in “the ATR Pathway and the Interplay between the DDR Network and the Immune System” [119]. Furthermore, Hsieh et al. (2022) observed ATR-mediated upregulation of CD47 and PD-L1 in irradiated colorectal cancer cells limits immune priming [120]. These findings suggest that ATR functions not only to maintain genomic stability but also to modulate tumor–immune interactions, highlighting its role as a central regulator linking DDR and adaptive immune responses. In our ATRi-resistance models, we observed downregulation of cytokine signaling (TNF-α, IFN-γ), alongside upregulation of pro-survival kinases in NF-κB pathway (CK2, RAF1) [121]. These changes were accompanied by evidence of widespread epigenetic shift particularly affecting IL-2/STAT5 and IL-6/STAT3 immune signaling pathways, suggesting that DNA methylation may serve as an adaptive mechanism contributing to compensatory cellular responses. These observations warrant further investigation to confirm their functional significance in future studies.

Beyond that, resistant ATRi-adaptation was characterized by extensive modulation of DNA repair networks, including HR repair, which represented the most promising therapeutic target among the downstream consequences of ATR inhibition identified in this study. On the genetic level, no distinct mutations were detected that could explain the modulation of compensatory immune or HR repair pathways. Instead, the observed epigenetic shift in DNA methylation, coupled with inverse transcriptomic profiles, argues for a heritable memory mechanism that maintains the ATRi-depleted expression networks and their phenotype. Indeed, we identified enriched pathways such as HR and BER involving DNA repair genes such as *POLQ*, *XRCC4*, and *BRCA1* potentially regulated by DNA methylation. Thus, the marked upregulation of DNA repair genes and associated DDR pathways in ATRi-resistant cells may hold vulnerabilities that, when targeted, could yield synergistic therapeutic effects by blocking critical bypass pathways. While, for instance, efficacy of PARP inhibitors was largely restricted to HR-deficient ovarian and breast tumors [122] recent studies proposed also a synergistic impact of ATR inhibitors to overcome PARP inhibitor resistance [123]. Moreover, accumulating studies propose promising effects of combined ATR and PARP inhibitor treatment in an *ATM*-deficient background [124–126].

However, *ATM* was not mutated in our patient-derived SCC and UC cultures, whereas the upregulation of HR repair factors such as *RAD51*, *BRCA1*, and *BRCA2* due to ATR loss suggested an increased reliance on HR repair to maintain genomic stability. This notion was supported by genomic profiling of the resistant cell lines, since these data revealed wild-type–comparable CNV status and TMB scores at lower levels, arguing for restored genetic stability and highlighting the crucial role of the ATR–HR axis. This hypothesis was confirmed by targeting the adaptive vulnerability using the RAD51 inhibitor B02. So far, several classes of RAD51i are under investigation that interfere with RAD51 function through distinct mechanisms, such as blocking oligomerization (IBR series: IBR2/120), preventing DNA binding (RI-1), or promoting protein degradation [127–129]. Among these, B02 remains the most widely used RAD51 inhibitor, known to sensitize tumor cells to radiation and chemotherapy while sparing normal cells, and has been broadly applied in preclinical models to study HR repair inhibition [130]. Moreover, derivatives of B02 (B02-iso, p-I-B02-iso) exhibit even higher potency and have been shown to sensitize triple-negative breast cancer (MDA-MB-231) cells to the PARP inhibitor Olaparib [128].

Here, in our ATRi-resistant models, we therapeutically demonstrated a notable re-sensitization following combined treatment with B02 and Ceralasertib, which blocked the HR–ATR axis. This re-sensitizing effect was evidenced by a marked increase in γH2AX foci and prolonged G2/M arrest upon IR exposure in p-SCC^ATRi^ models. Moreover, the combination treatment significantly stunted tumor growth, with a markedly increased amount of apoptosis in *in ovo* xenografts. Krajewska and colleagues already proposed a therapeutic connection between HR repair and ATR inhibition in 2015 [54]. This study modulated genomic instability by RAD51 inactivation revealing a therapeutic vulnerability at this level, which was targetable with ATRi, i.e. the same connection we found but the other way around. However, apart from that and to our best knowledge, the axis has not been studied so far. Our findings provide first functional evidence of a reciprocal relationship between ATR and HR repair in BLCA underscoring the potential for therapeutically exploiting this connection, particularly for the rare and therapeutic challenging patient group diagnosed with squamous differentiated tumors.

Since short-term inactivation of ATR triggers HR repair factor expression within days, our findings argue for compensatory HR repair activation through RAD51 as consequence of a direct feedback loop between ATR and HR repair. In turn, given the DNA methylation shift involving HR repair components as well, our data suggest an epigenetic memory impact on DDR gene regulation in response to ATR loss that is potentially bridged by more flexible histone modification processes, as suggested by previous study [131]. In that study, Thy et al. demonstrated an influence of bromodomain (BRD) inhibitor PLX51107 on DNA repair gene expression including *RAD51* and *BRCA1*. This observation could provide clues for further novel therapeutic strategies – such as epidrugs as small molecules targeting epigenetic pathways – and at the same time strengthens the rationale for combined ATRi–RAD51i treatment in patient groups with ATR deficiency.

## Conclusion

In this study, we demonstrated that ATRi Ceralasertib exhibits potent radio-sensitizing effects in patient-derived ex vivo UC cell cultures and especially in SCC cell cultures. Furthermore, ATRi-adaptations associated with a kind of epigenetic reprogramming could be effectively counteracted by targeting underlying compensatory repair processes, specifically through RAD51 inhibition. Thus, targeting ATR may help to develop multifaceted treatment strategies. Given the genetic landscape suggesting a putative ATR- and HR-deficiency in BLCA, our findings provide a strong rationale for the clinical evaluation of ATR inhibitors as radio-sensitizers, as well as for their combination with RAD51 inhibitors to exploit synthetic vulnerabilities in future therapeutic strategies.

## Supplementary Information

Supplementary Material 1

Supplementary Material 2

## Data Availability

All data that were generated or analyzed during our study have been included in this article. Materials, additional data and protocols described within the manuscript will be made available from the authors upon reasonable request. The RNA-Seq data reported in this study are deposited in the GEO repository with accession number GSE301614.

## Abbreviations

ATR: Ataxia telangiectasia and Rad3 related kinase
ATRi: Ataxia telangiectasia and Rad3 related kinase inhibitor
ATR^bypass^: ATRi-resistant with HR activation
ATR^mut^: ATR-mutated
BER: Base excision repair
BLCA: Bladder cancer
BRCA1/2: Breast cancer type 1/2
CAM: Chorioallantoic membrane
CC-3: Cleaved caspase 3
*CHEK1*/CHK1: Checkpoint kinase 1 (gene/protein)
CK5/6/7: Cytokeratin 5/6/7 (protein)
CNV: Copy number variation
DDR: DNA damage response
DEG: Differentially expressed genes
DSB: Double-strand break
ED: Embryonic day
FA: Fanconi anemia
FACS: Fluorescence-activated cell sorting
FFPE: Formalin-fixed, paraffin-embedded
GAPDH: Glyceraldehyde-3-phosphate dehydrogenase
GATA3: GATA binding protein 3
GO: Gene ontology
GRCh38: Genome reference consortium human build 38
GSEA: Gene set enrichment analysis
GSVA: Gene Set Variation Analysis
Gy: Gray (unit of radiation dose)
HE: Hematoxylin and eosin
HR: Homologous recombination
HRD: Homologous recombination repair deficiency
IC50: Half maximal inhibitory concentration
IF: Immunofluorescence
IHC: Immunohistochemistry
IR: Ionizing radiation
IRS: Immunoreactive score
KEGG: Kyoto encyclopedia of genes and genomes
KRT5/6/7: Cytokeratin 5/6/7(gene)
MIBC: Muscle-invasive bladder cancer
MMR: Mismatch repair
MSigDB: Molecular Signatures Database
NER: Nucleotide excision repair
NHEJ: Non-homologous end joining
OS: Overall survival
p-SCC: Patient-derived ex vivo cell culture of SCC
p-SCC^ATRi^: ATR inhibitor adapted patient-derived ex vivo cell culture of SCC
p-UC: Patient-derived ex vivo cell culture of UC
p53/*TP53*: Tumor suppressor protein p53 (protein/gene)
PARP: Poly(ADP-ribose) polymerase
PDC: Patient-derived ex vivo culture
PI: Propidium iodide
PTK: Peptide tyrosine kinase
RAD51: Rad51 recombinase
RAD51i: Rad51 recombinase inhibitor
RC: Radical cystectomy
RFS: Relapse-free survival
RNA-Seq: RNA sequencing
SCC: Squamous cell carcinoma
SD: Standard deviation
SEM: Standard error of the mean
STK: Serine/threonine kinase
TCGA: The Cancer Genome Atlas
TMB: Tumor mutational burden
TME: Tumor microenvironment
TMT: Trimodality therapy
TURBT: Transurethral resection of bladder tumor
UC: Urothelial carcinoma
VUS: Variant of uncertain significance
WES: Whole exome sequencing
γH2AX: Phosphorylated histone H2AX
