# Supplementary material for: Targeting ATR offers multifaceted treatment strategies involving RAD51-mediated compensatory DNA repair in bladder cancer
Source: J Exp Clin Cancer Res. 2025 Dec 12;45:17. doi: 10.1186/s13046-025-03603-4 (PMC12817416; doi:10.1186/s13046-025-03603-4)
Supplement: Supplementary file 1 — Supplementary Material 1 [file 13046_2025_3603_MOESM1_ESM.docx]

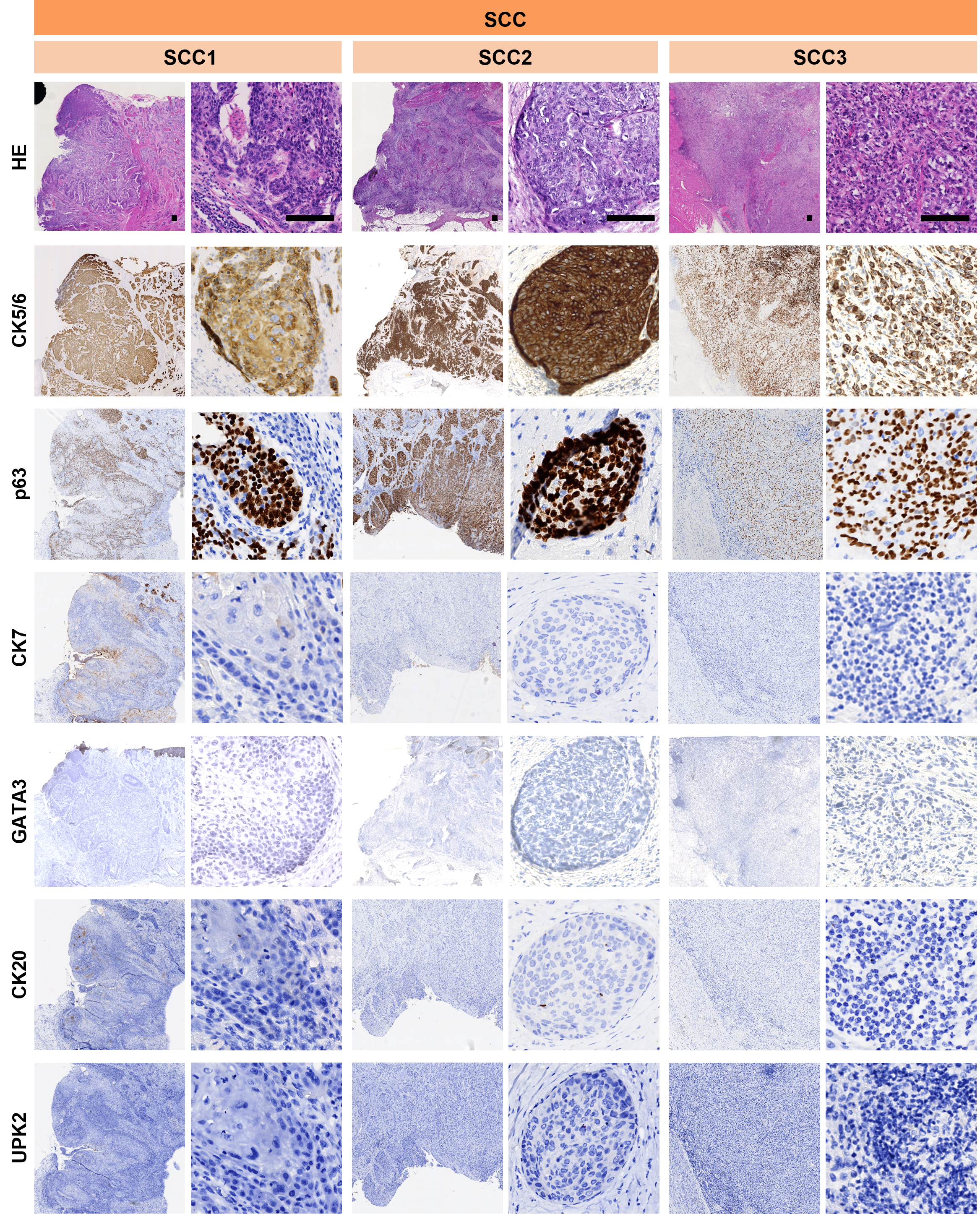


**Supplementary Figure S1: Histopathological and immunohistochemical characterization of SCC tumor tissues.** Supplement to main Figure 1. Representative images of HE staining and immunohistochemical analyses (CK5/6, p63, CK7, GATA3, CK20, UPK2) of parental tumor tissues from three squamous cell carcinomas (SCC1, SCC2, SCC3) used in this study. For each tumor and each staining, representative regions are shown at low (left) and high (right) magnification (scale bar: 100 µm).


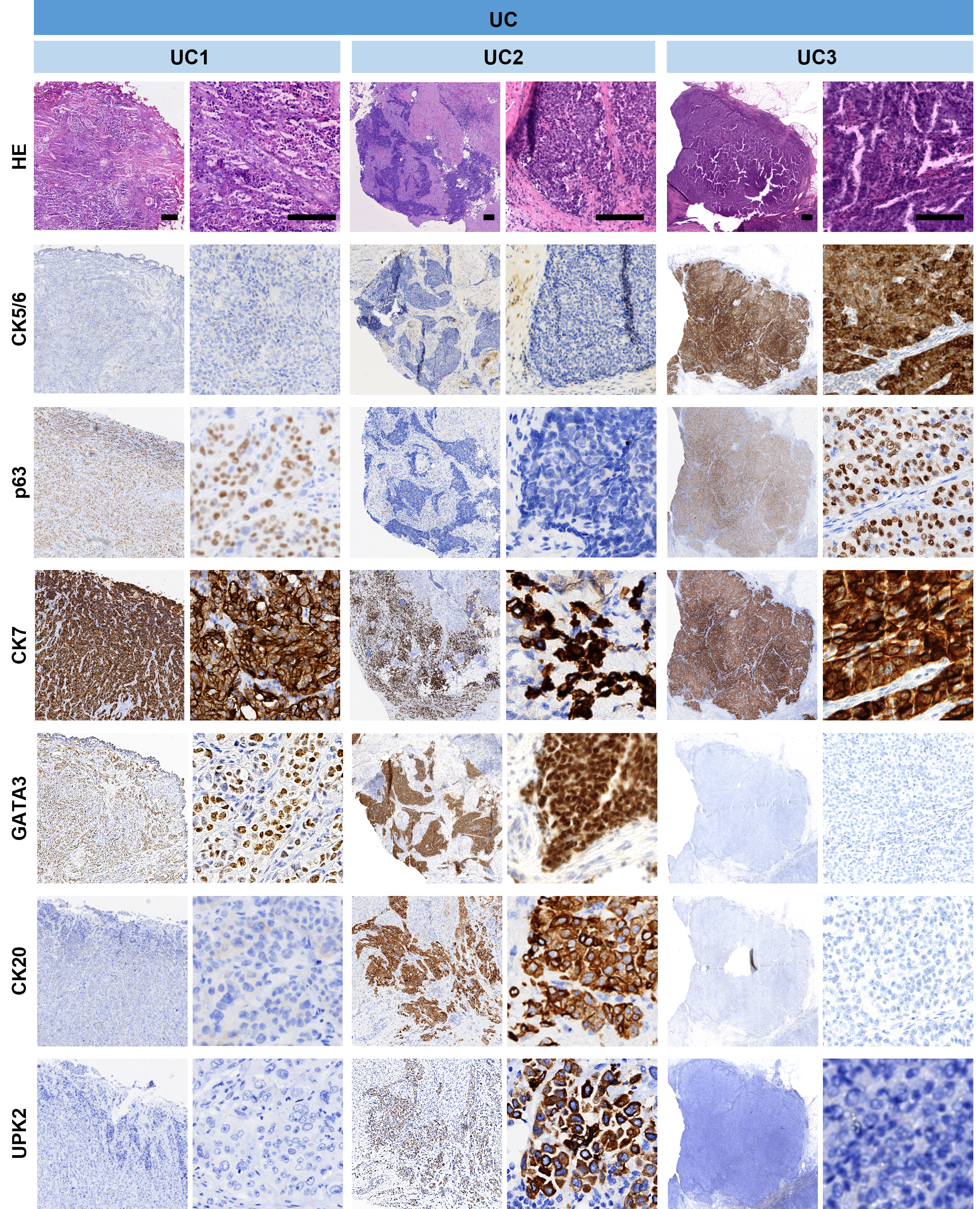


**Supplementary Figure S2: Histopathological and immunohistochemical characterization of UC tumor tissues.** Supplement to main Figure 1. Representative images of HE staining and immunohistochemical analyses (CK5/6, p63, CK7, GATA3, CK20, UPK2) of parental tumor tissues from three urothelial carcinomas (UC1, UC2, UC3) used in this study. For each tumor and each staining, representative regions are shown at low (left) and high (right) magnification (scale bar: 100 µm).

**
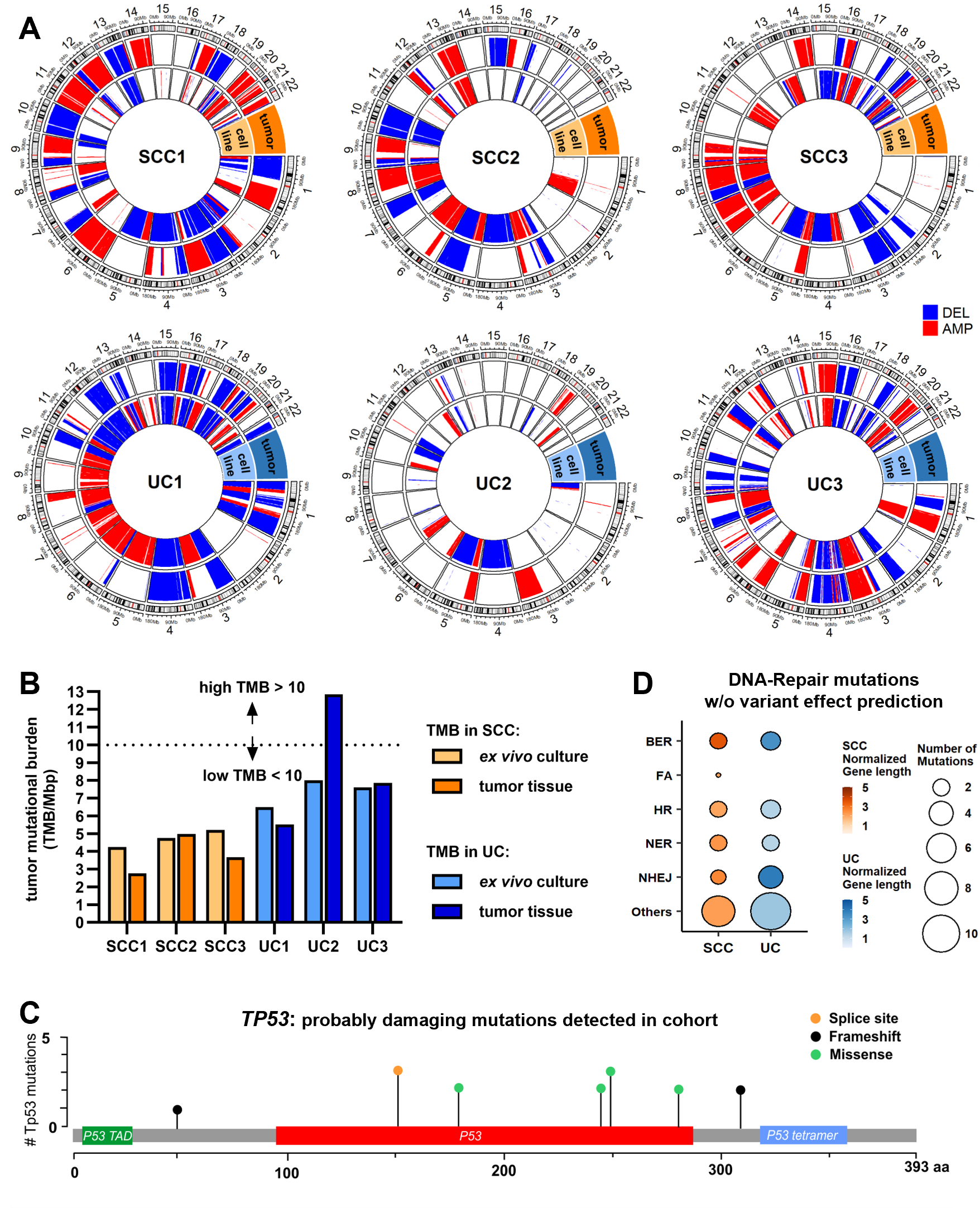
**

**Supplementary Figure S3: Mutational landscape of SCC and UC models.** Supplement to main Figure 1. **(A)** Copy number analysis (WES) comparing SCC tumors with matched ex vivo cultures (p‑SCC, top) and UC tumors with p‑UC cultures (bottom). Inner ring: cell line; outer ring: tumor; blue = deletion; red = amplification. **(B)** TMB analysis of tumor tissues (SCC, dark orange; UC, dark blue) and corresponding cell lines (p‑SCC, light orange; p‑UC, light blue). **(C)** Lollipop plot of “probably damaging” TP53 mutations in all tumors and cell lines. Functional domains: transactivation (green), DNA-binding (red), tetramerization (blue). **(D)** Reactome pathway dot plot showing DNA repair–related mutations (unfiltered for variant effect; NA, uncertain, probably damaging, probably benign) in SCC- (orange) and UC-associated (blue) samples (n = 6).


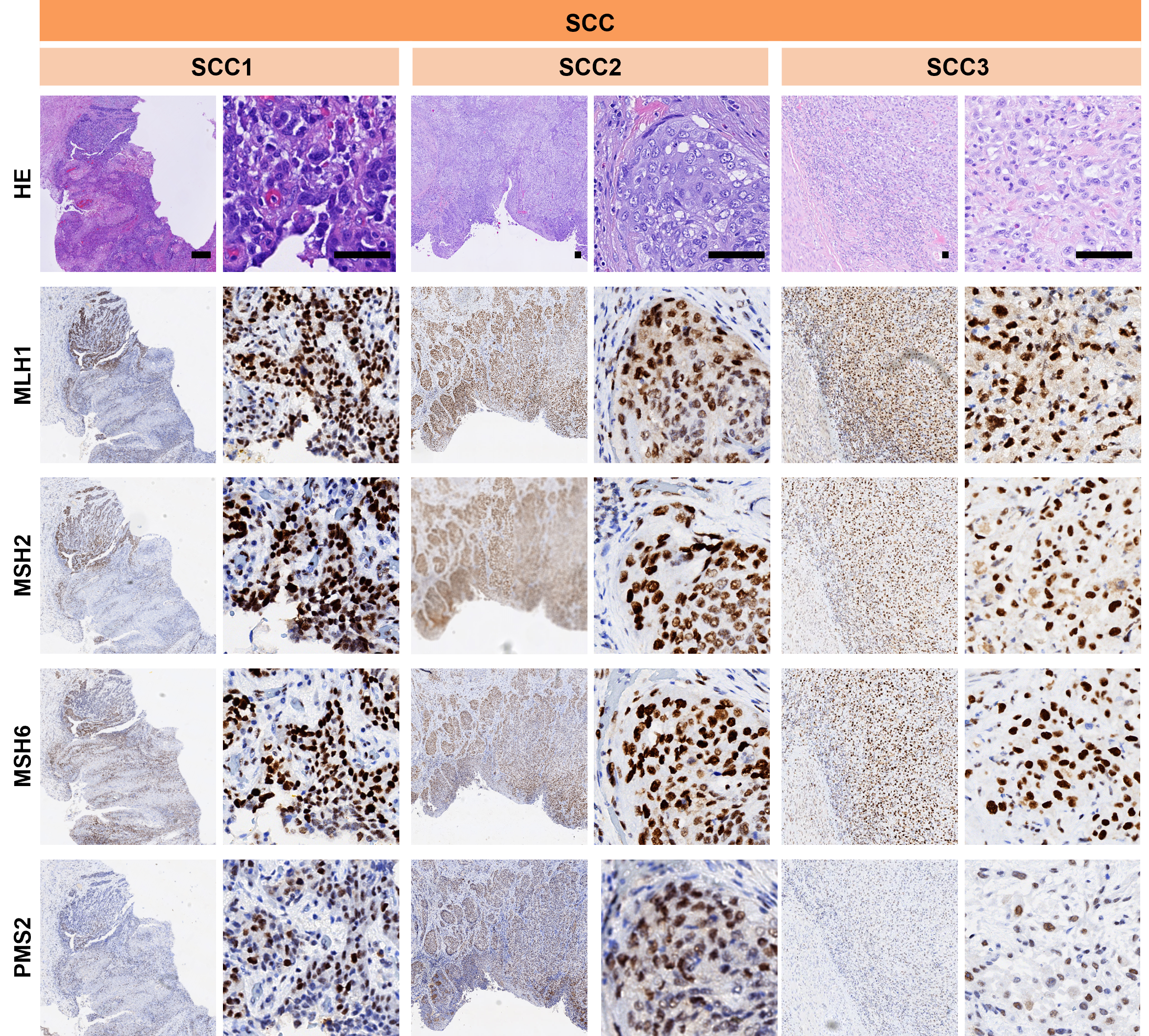


**Supplementary Figure S4: Immunohistochemical characterization of SCC tumor tissues for microsatellite stability.** Supplement to main Figure 1. Representative images of HE staining and immunohistochemical analyses (MLH1, MSH3, MSH6, PMS2) of parental tumor tissues from three squamous cell carcinomas (SCC1, SCC2, SCC3) used in this study. For each tumor and each staining, representative regions are shown at low (left) and high (right) magnification (scale bar: 100 µm).


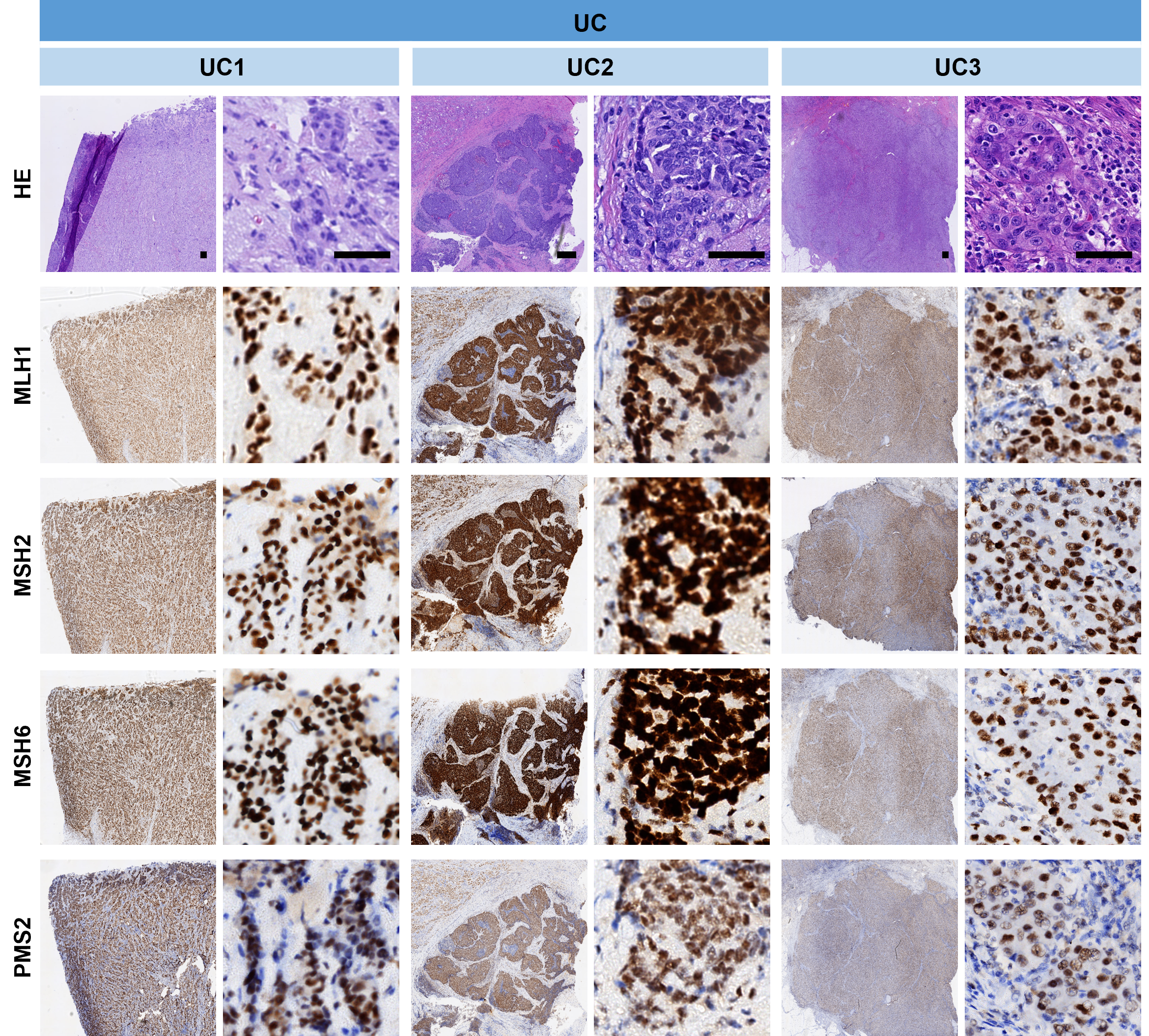


**Supplementary Figure S5: Immunohistochemical characterization of UC tumor tissues for microsatellite stability.** Supplement to main Figure 1. Representative images of HE staining and immunohistochemical analyses (MLH1, MSH3, MSH6, PMS2) of parental tumor tissues from three urothelial carcinomas (UC1, UC2, UC3) used in this study. For each tumor and each staining, representative regions are shown at low (left) and high (right) magnification (scale bar: 100 µm).


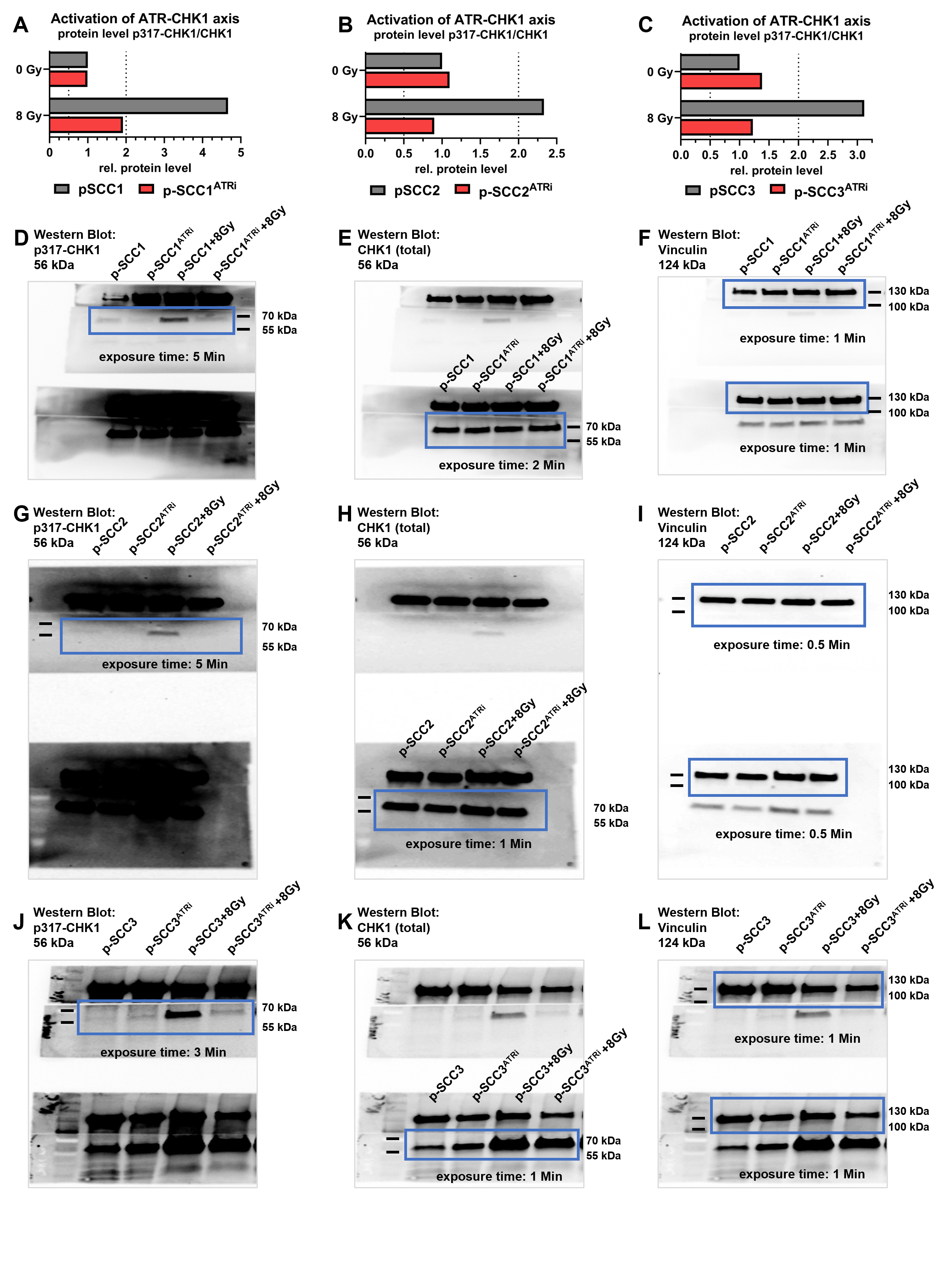


**Supplementary Figure S6: Protein-level analysis and raw immunoblot images related to main Figure 4F–H. (A-C)** Protein-level analysis showing activation of the ATR–CHK1 axis in (A) p-SCC1/p-SCC1^ATRi^, (B) p-SCC2/p-SCC2^ATRi^ and (C) p-SCC3/p-SCC3^ATRi^ cell lines by densitometric quantification of immunoblot bands. (D–L) Raw immunoblot images. Blue boxes highlight the relevant bands for (D, G, J) pS317‑CHK1 (56 kDa), (E, H, K) total CHK1 (56 kDa), and (F, I, L) loading control Vinculin (124 kDa) in (D-F) p-SCC1/p-SCC1^ATRi^, (G-I) p-SCC2/p-SCC2^ATRi^ and (J-L) p-SCC3/p-SCC3^ATRi^ cell lines with the indicated exposure times. Activation was calculated as the ratio pS317‑CHK1/total CHK1 relative to Vinculin via ImageJ.


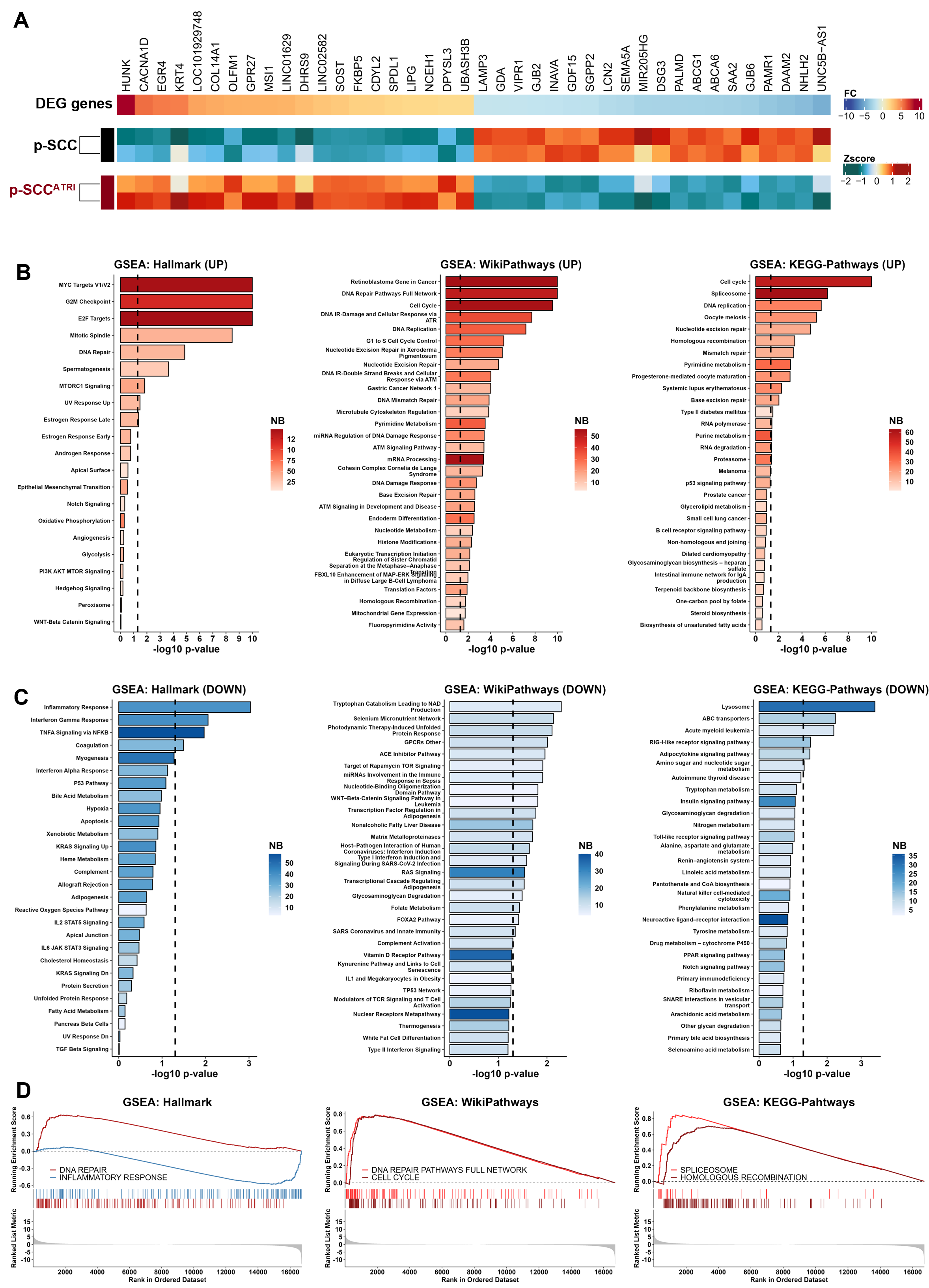


**Supplementary Figure S7: Transcriptomic and GSEA pathway-level effects of ATR inhibition in p-SCC cells.** Supplement to main Figure 5. **(A)** Heatmap of differentially expressed genes (DEGs) in p‑SCC^ATRi^ versus untreated p‑SCC control. Expression values were scaled per gene across samples. Gene Set Enrichment Analysis (GSEA) for **(B)** upregulated and **(C)** downregulated pathways in p-SCC^ATRi^. Bar plots show the top 30 enriched terms from the Hallmark, WikiPathways, and KEGG sets. **(D)** Enrichment plots (gseaplot2) for selected Hallmark, WikiPathways, and KEGG gene sets showing the running enrichment score (ES), gene rank distribution, and core enrichment genes.


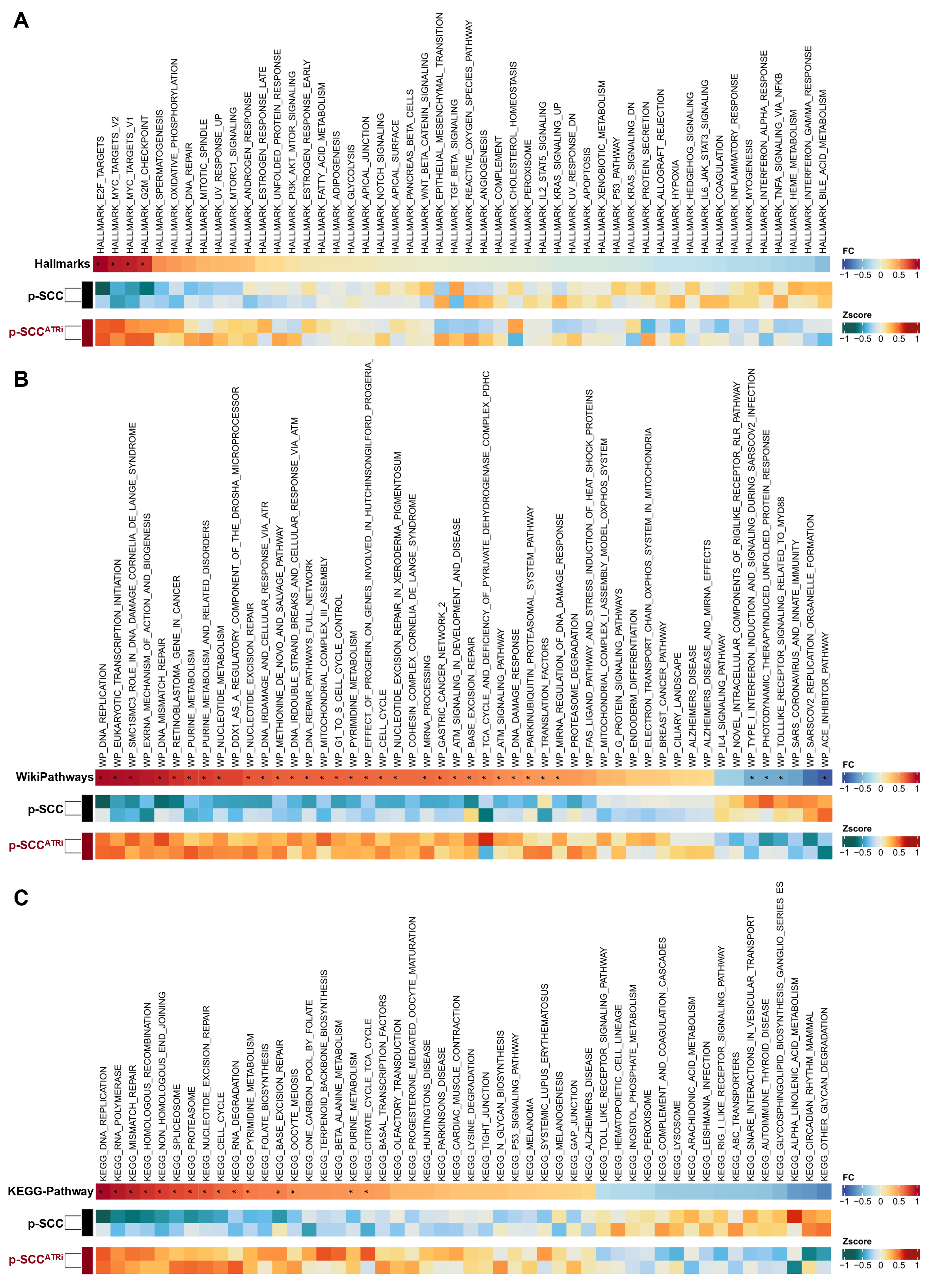


**Supplementary Figure S8: Transcriptomic GSVA pathway-level effects of long-term ATR inhibition in p‑SCC cells.** Supplement to main Figure 5. Gene Set Variation Analysis (GSVA) in p‑SCC^ATRi^ versus untreated p‑SCC control showing **(A)** Hallmarks, **(B)** WikiPathways, and **(C)** KEGG pathways. Significant pathways (FC > 0.5; p < 0.05) are indicated by asterisks.


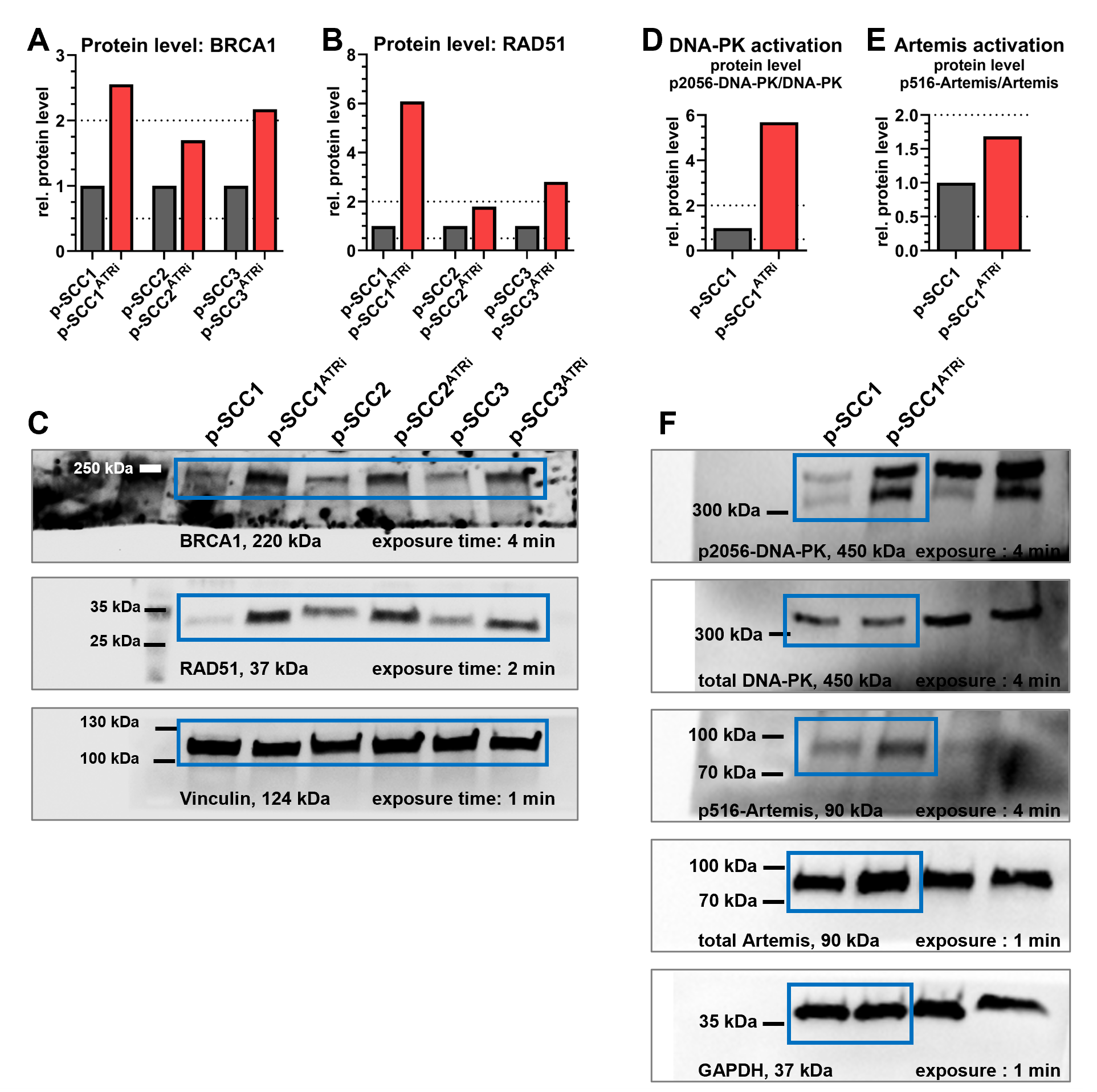


**Supplementary Figure S9: HR and NHEJ factor validation at the protein level.** Supplement to main Figure 7. **(A–C)** Protein-level analysis related to Figure 7A showing BRCA1 (A) and RAD51 (B) expression in p-SCC1/p-SCC1^ATRi^, p-SCC2/p-SCC2^ATRi^ and p-SCC3/p-SCC3^ATRi^ cell lines via immunoblots. Relative levels were normalized to Vinculin (C) Raw immunoblot images. Blue boxes mark bands for BRCA1 (220 kDa), RAD51 (37 kDa), and Vinculin (124 kDa) with indicated exposure times. **(D–F)** Protein-level analysis of additional NHEJ factors showing (D) DNA‑PK and (E) Artemis activation in p-SCC1/p-SCC1^ATRi^ cell lines by immunoblot quantification. Activation was calculated as the ratio pS2056‑DNA‑PK/total DNA‑PK and pS516‑Artemis/total Artemis normalized to GAPDH via ImageJ. (F) Raw immunoblot images. Blue boxes highlight bands for pS2056-DNA-PK (450 kDa), total DNA‑PK (450 kDa), pS516-Artemis (90 kDa), total Artemis (90 kDa), and GAPDH (37 kDa). (n = 1)


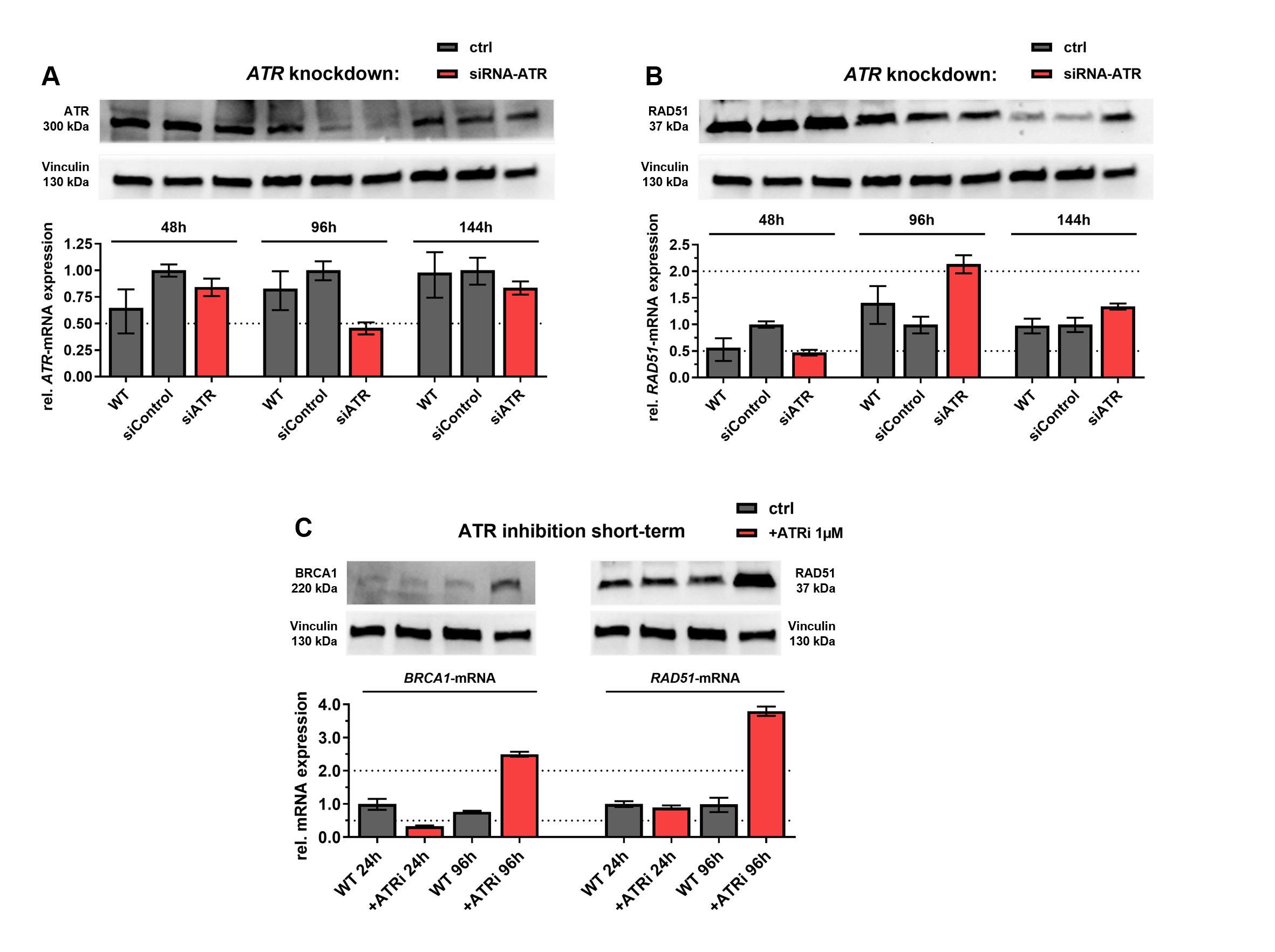


**Supplementary Figure S10: HR compensation linked to feedback loop of ATR loss.** Supplement to main Figure 7. **(A–B)** Transient ATR knockdown in p‑SCC1 wild-type cells (WT) using siRNA. (A) ATR protein (top) and mRNA (bottom) levels were measured 48 h, 96 h, and 144 h post-transfection. (B) RAD51 protein (top) and mRNA (bottom) levels at the same time points. Samples were normalized to siControl. **(C)** Short-term ATR inhibition in p‑SCC1 cells treated with 1 µM Ceralasertib for 24 h or 96 h. Samples were normalized to 24 h WT. Red indicates ATR-depleted conditions; gray indicates controls (n = 1).
